# The reaction of arylmethyl isocyanides and arylmethylamines with xanthate esters: a facile and unexpected synthesis of carbamothioates

**DOI:** 10.3762/bjoc.16.18

**Published:** 2020-02-03

**Authors:** Narasimhamurthy Rajeev, Toreshettahally R Swaroop, Ahmad I Alrawashdeh, Shofiur Rahman, Abdullah Alodhayb, Seegehalli M Anil, Kuppalli R Kiran, Chandra, Paris E Georghiou, Kanchugarakoppal S Rangappa, Maralinganadoddi P Sadashiva

**Affiliations:** 1 Department of Studies in Chemistry, University of Mysore, Manasagangothri, Mysuru, Karnataka 570 006, India; 2 Department of Chemistry, Memorial University of Newfoundland, St. John’s, Newfoundland and Labrador A1B3X7, Canada; 3 Aramco Laboratory for Applied Sensing Research, King Abdullah Institute for Nanotechnology, King Saudi University, Riyadh, Saudi Arabia; 4 Surface, and Interface Sciences, Department, of Physics and Astronomy, College of Science, King Saudi University, Riyadh 11451, Saudi Arabia; 5 Department of Physics, National Institute of Engineering, Mysuru, Karnataka 570 008, India

**Keywords:** benzylamines, carbamothioates, density functional theory, intrinsic reaction coordinate analysis, isocyanides, sodium hydride, xanthate esters

## Abstract

An unexpected formation of carbamothioates by a sodium hydride-mediated reaction of arylmethyl isocyanides with xanthate esters in DMF is reported. The products thus obtained were compared with the carbamothioates obtained by the sodium hydride-mediated condensation of the corresponding benzylamines and xanthate esters in DMF. To account for these unexpected reactions, a mechanism is proposed in which the key steps are supported by quantum chemical calculations.

## Introduction

Carbamothioates (thiocarbamates) have been reported to have antimicrobial [1], antifungal (e.g., tolnaftate and tolciclate) [2], antimycobacterial [3], human leucocyte elastase inhibitory [4], TRPV1 antagonistic [5], and PPARα1γ dual antagonistic [6] properties, and also act as intermediates in the syntheses of HIV-1 integrase ligands [7], insecticides (cartap) [8], and herbicides [9]. They are also used as key intermediates in the generation of carbonyl sulfide/hydrogen sulfide [10], the synthesis of isothiocyanates [11], asymmetric thioureas [12], and thiazolidine/thiaoxazine [13]. Therefore, as a result, numerous synthetic methods for carbamothioates have been reported. These include reactions of chlorothioformates with amines [14], thiocarbonyl benzotriazoles with alcohols [15], copper-catalyzed reactions of α-substituted stannanes with carbamothioates [16], reactions of isothiocyanates with alcohols [6,17], and reactions of xanthate esters with amines [18]. Furthermore, many methods have also been reported for the synthesis of cyclic thiocarbamates, and these include reactions of isothiocyanates with aldehydes in the presence of organocatalysts [19–20], reactions of vicinal diols with potassium thiocyanate [21], iron nanoparticle-catalyzed reactions of 2-naphthol with benzaldehyde and some of its derivatives with thiourea [22], isothiocyanato oxindoles with ketones [23], ammonium isothiocyanates with chalcones [24], and α-isothiocyanato esters with α-keto amides [25]. Among the synthetic methods available for the synthesis of open-chain thiocarbamates, however, many suffer from limitations, such as the use of less stable and sensitive reactants, for example, chlorothioformates [6,14,16–17], toxic stannates [16], and isothiocyanates. In a single patent disclosure, thiocarbamates were reported to have been synthesized from xanthate esters, but the methodology described is limited to only a few examples with aliphatic substituents and furthermore suffers from a tedious isolation protocol [18].

**Scheme 1:**
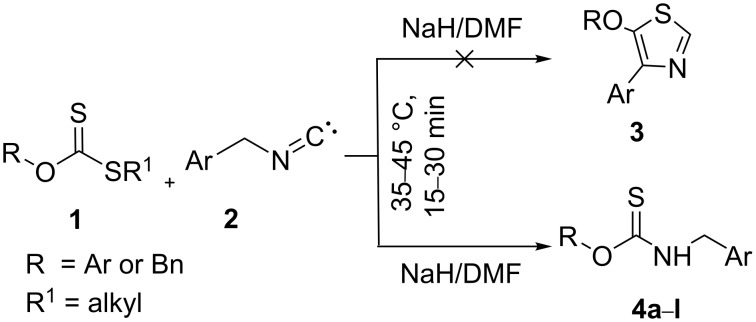
Synthesis of carbamothioates from xanthate esters and benzyl isocyanides.

As a part of our work on the development of new synthetic methods [26–30], we have recently reported the synthesis of thiazoles from xanthate esters [31]. In continuation of this ongoing work, we planned to synthesize 5-alkoxy-4-arylthiazoles **3** by the sodium hydride/DMF-mediated reaction of arylmethyl isocyanides **2** with *S*-alkyl xanthate esters **1** or *O*-aryl/*O*-alkyl dithiocarbonates. Unexpectedly, however, carbamothioates **4a**–**l** were instead obtained in 76–88% yield (Scheme 1). Herein, we report on this intriguing finding and show several examples, including a single crystal X-ray structure of one of the products so obtained. A plausible mechanism to explain the reaction using density functional theory (DFT) analysis is also presented in this article.

## Results and Discussion

### Synthesis

At the onset of our study, the reaction between *O*-benzyl *S*-methyl dithiocarbonate (**1a**) and benzyl isocyanide was conducted in the presence of sodium hydride in DMF. The product, obtained in 85% yield after 10 min (Table 1, method A, entry 1), was unexpectedly found to be *O*-benzyl benzylcarbamothioate (**4a**). The spectral data indicated that the product existed in *cis*- and *trans*-geometrical isomeric forms (rotamers) because of free rotation along the thioamide bond. When the same reaction was conducted in other solvents, such as THF, acetonitrile, dioxane, DMSO, or toluene, in the presence of sodium hydride, none of these reactions afforded the product **4a** in a satisfactory yield (Table 1, method A, entries 2–6). Replacement of sodium hydride by DBU did not furnish any product at all (Table 1, method A, entry 7), and a 50% reduction in the quantity of sodium hydride did not affect the yield (Table 1, method A, entry 8). Notably, however, the use of only a catalytic amount of sodium hydride also failed to afford any product.

**Table 1:** Optimization data for the synthesis of **4a**.

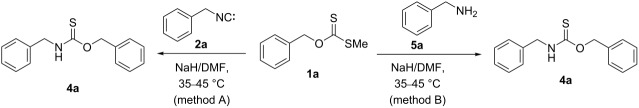				

method A^a^				
entry	solvent	base	time	yield of **4a**, %

1	DMF	NaH^a^	10 min	85
2	THF	NaH^a^	4 h	45
3	CH_3_CN	NaH^a^	3 h	53
4	dioxane	NaH^a^	4 h	48
5	DMSO	NaH^a^	2 h	58
6	toluene	NaH^a^	24 h	10
7	DMF	DBU	10 h	0
8	DMF	NaH^c^	15 min	83
9	DMF	NaH^d^	24 h	0

method B^b^				
entry	solvent	base	time	yield of **4a**, %

1	DMF	NaH	1 h	80
2	THF	NaH	6 h	35
3	CH_3_CN	NaH	6 h	55
4	DMSO	NaH	3 h	58
5	toluene	NaH	12 h	29
6	DMF	NaH^c^	1 h	74
7	DMF	DBU	24 h	0

^a^Reaction conditions: *O*-benzyl *S*-methyl dithiocarbonate (**1a**, 1.0 mmol), benzyl isocyanide (**2a**, 1.0 mmol), NaH (2.0 mmol), DMF (2.0 mL), 35–45 °C. ^b^Reaction conditions: **1a** (1.0 mmol), **5a** (1.0 mmol), NaH (2.0 mmol), DMF (2.0 mL), 30–40 °C. ^c^NaH (1.0 mmol) was used. ^d^A catalytic amount of 5 mol % NaH was used.

**Figure 1:**
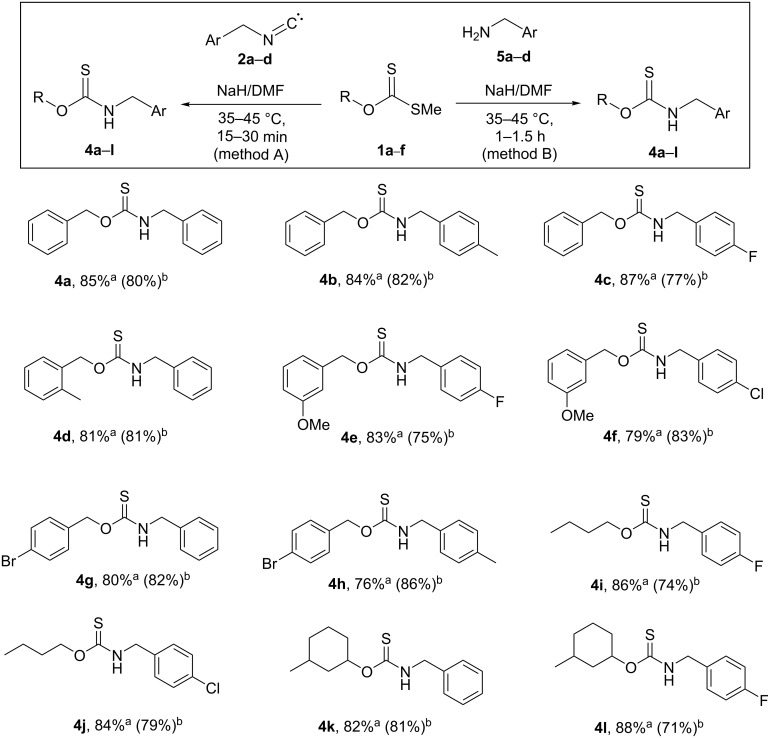
Substrate scope for the synthesis of carbamothioates. Reaction conditions for methods A and B: sodium hydride (2.0 mmol), **1a**–**f** (1.0 mmol), **2** or **5** (1.0 mmol), DMF (2.0 mL). ^a^Isolated yield from method A. ^b^Isolated yield from method B.

Using the optimized reaction conditions that were established for **4a**, the reactions of **1a** with 4-methylbenzyl isocyanide (**2b**) and 4-fluorobenzyl isocyanide (**2c**) gave the corresponding products **4b** and **4c** in 84% and 87% yield, respectively (Figure 1). *S*-Methyl *O*-(2-methylbenzyl) dithiocarbonate (**1b**) reacted with benzyl isocyanide (**2a**) to give *O*-(2-methylbenzyl) benzylcarbamothioate (**4d**) in 81% yield. *O*-(3-Methoxybenzyl) *S*-methyl dithiocarbonate (**1c**) reacted with 4-fluorobenzyl isocyanide (**2c**) or 4-chlorobenzyl isocyanide (**2d**) to give the corresponding carbamothioates **4e** and **4f** in 83% and 79% yield, respectively. The generality of the reaction was further probed by reacting *O*-(4-bromobenzyl) *S*-methyl dithiocarbonate (**1d**) with benzyl isocyanide (**2a**) and 4-methylbenzyl isocyanide (**2b**), which afforded the corresponding carbamothioates **4g** and **4h** in 80% and 76% yield, respectively. Interestingly, with *O*-butyl *S*-methyl dithiocarbonate (**1e**), the xanthate ester synthesized from *n*-butanol, the corresponding *O*-butyl (4-fluorobenzyl)carbamothioate (**4i**) and *O*-butyl (4-chlorobenzyl)carbamothioate (**4j**), were produced when reacted with 4-fluorobenzyl isocyanide (**2c**) and 4-chlorobenzyl isocyanide (**2d**) in similar yields of 86% and 84%, respectively. Finally, *S*-methyl *O*-(3-methylcyclohexyl) dithiocarbonate (**1f**) also afforded the corresponding carbamothioates **4k** and **4l** in 82% and 88% yields, with benzyl isocyanide (**2a**) and 4-fluorobenzyl isocyanide (**2c**), respectively. The use of a weaker base, such as DBU, failed to form any product (Table 1, methods A and B, entry 7).

For the purpose of comparison, the condensation reaction of *O*-benzyl *S*-methyl dithiocarbonate (**1a**) with benzylamine **5a** in the presence of sodium hydride as the base of choice was evaluated using different solvents, DMF, THF, acetonitrile, DMSO, and toluene (Table 1, method B, entries 1–5). DMF was found to be the best solvent, yielding *O*-benzyl benzylcarbamothioate (**4a**) in 80% yield after 1 h (Table 1, method B, entry 1). A decreased amount of base reduced the yield slightly (Table 1, method B, entry 6). The versatility of the synthetic methodology was further investigated by reacting *O*-benzyl *S*-methyl dithiocarbonate (**1a**) with 4-methylbenzylamine (**5b**) and 4-fluorobenzylamine (**5c**), which respectively yielded *O*-benzyl (4-methylbenzyl)carbamothioate (**4b**) and *O*-benzyl (4-fluorobenzyl)carbamothioate (**4c**) in 82% and 77% yield (Figure 1).

**Figure 2:**
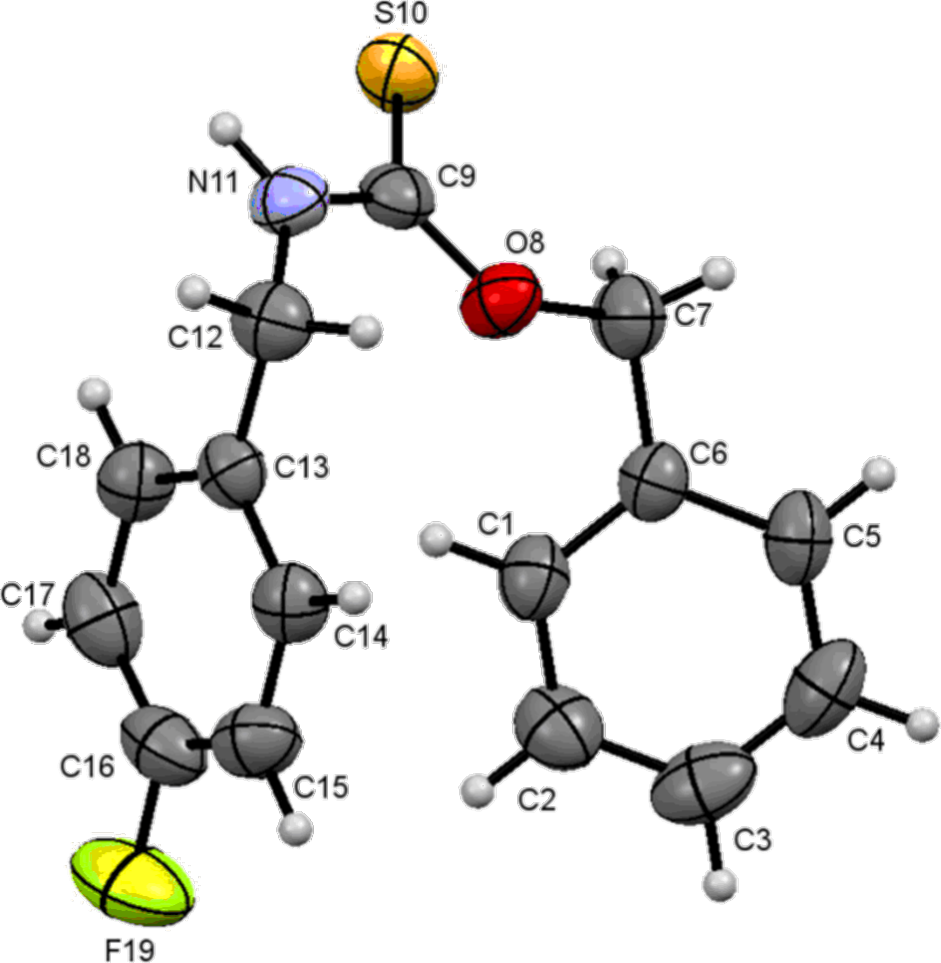
ORTEP diagram of *O*-benzyl (4-fluorobenzyl)carbamothioate (**4c**).

**Figure 3:**
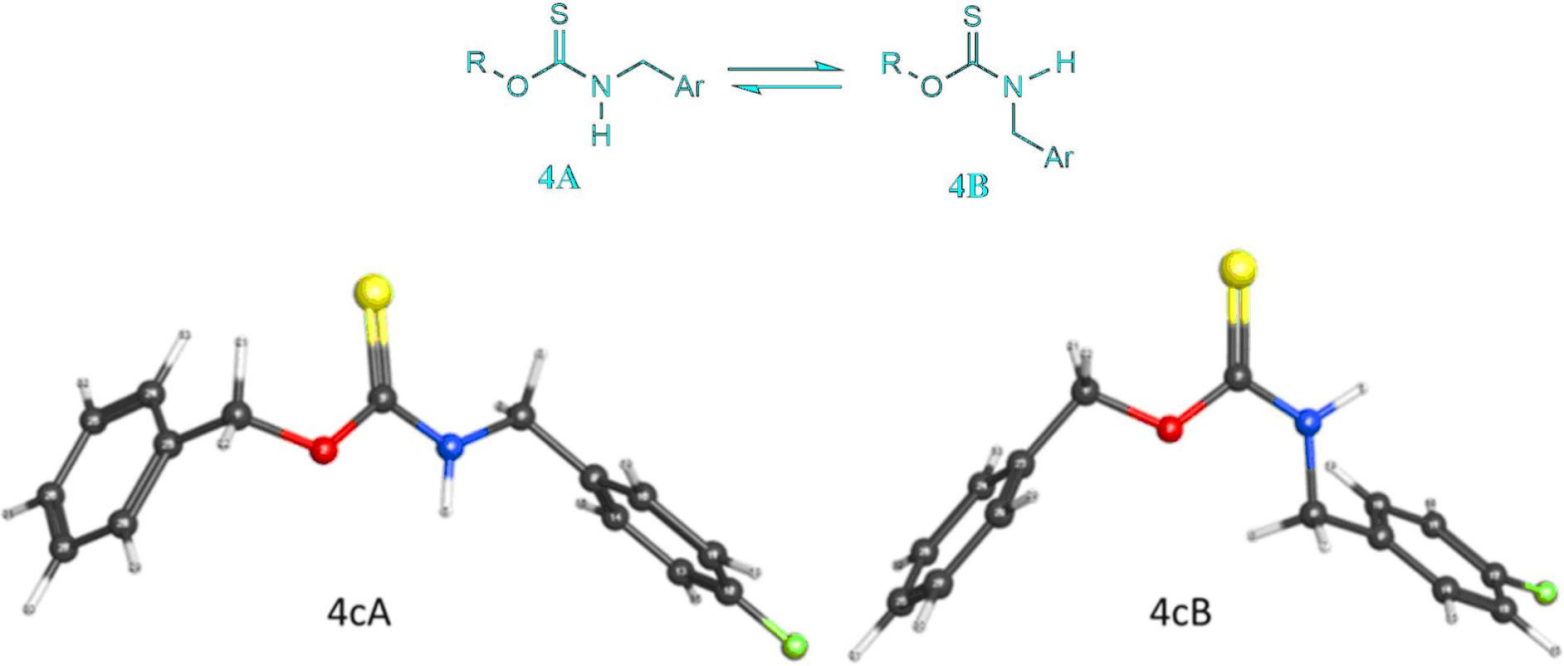
Rotamers of thionocarbamates **4** (top) and computer-minimized structures of **4c** (bottom).

*S*-Methyl *O*-(2-methylbenzyl) dithiocarbonate (**1b**) reacted smoothly with benzylamine (**5a**) to give *O*-(2-methylbenzyl) benzylcarbamothioate (**4d**) in 81% yield. The xanthate ester *O*-(3-methoxybenzyl) *S*-methyl carbonodithioate (**1c**) underwent condensation with 4-fluorobenzylamine (**5c**) and 4-chlorobenzylamine (**5d**) to afford the corresponding *O*-(3-methoxybenzyl) (4-fluorobenzyl)carbamothioate and *O*-(3-methoxybenzyl) (4-chlorobenzyl)carbamothioate **4e** and **4f** in 75% and 83% yield, respectively. The xanthate ester *O*-(4-bromobenzyl) *S*-methyl dithiocarbonate (**1d**) also reacted successfully with benzylamine (**5a**) and 4-methylbenzylamine (**5b**) to furnish the corresponding carbamothioates **4g** and **4h** in 82% and 86% yield, respectively. *O*-Butyl *S*-methyl dithiocarbonate (**1e**), the xanthate ester derived from the aliphatic alcohol *n*-butanol, also gave the corresponding *O*-butyl (4-fluorobenzyl)carbamothioate and *O*-butyl (4-chlorobenzyl)carbamothioate **4i** and **4j** in 74% and 79% yield, respectively, from the reactions with 4-fluorobenzylamine (**5c**) and 4-chlorobenzylamine (**5d**). Finally, the cycloalkyl xanthate ester *S*-methyl *O*-(3-methylcyclohexyl) carbonodithioate (**1f**) also underwent a condensation with benzylamine (**5a**) and 4-fluorobenzylamine (**5c**) to give carbamothioates **4k** and **4l** in 81 and 71% yield, respectively. The NMR spectra of the carbamothioate products obtained indicated that, apart from **4e** and **4f**, all existed as rotamers and that the ratios of rotamers, where present, were the same whether derived from either method A or B. Alajarin et al. [32] noted a similar doubling of ^1^H and ^13^C NMR signals due to rotamers in one of their *O-*benzyl *N*-thiocarbamates. The structure of one of the carbamothioates, **4c**, was confirmed by a single crystal X-ray diffraction study (Figure 2 as well as Tables S1 and S2, Supporting Information File 1, CCDC reference number: 1831389) [33]. A DFT modeling study was then conducted at the B3LYP/6-311++G(d,p) level of theory, with solvent corrections for chloroform, for two rotamers, namely **4cA** and **4cB** (generically represented as **4A** and **4B** in Figure 3). These structures were generated based on the X-ray structure of **4c** and afforded a computed Gibbs free energy difference of −1.769 kJ mol^−1^ in favor of **4cB**. The resulting calculated equilibrium constant of 2.042, corresponding to a 67.1/32.9 ratio of the rotamers (**4cB**/**4cA**), was in good agreement with the experimentally by ^1^H NMR (CDCl_3_) observed ratio of 65/35. Significantly, the single crystal of **4c**, which afforded the crystal structure shown in Figure 3, corresponds to rotamer **4cB**. A limited variable-temperature ^1^H NMR study was conducted by heating a solution of **4c** in DMSO-*d*_6_ from ambient temperature up to 60 °C, but no changes were observed in the ratio of the rotamers.

We initially hypothesized that the isocyanides could have undergone a reductive cleavage to give the corresponding benzylamines, which might have reacted with **1** to give **4**. A control experiment was therefore conducted with only the isocyanide under standard reaction conditions. Only unchanged isocyanide was found under these conditions, thus ruling out this initial hypothesis.

### Computational studies on the proposed reaction mechanism

**Scheme 2:**
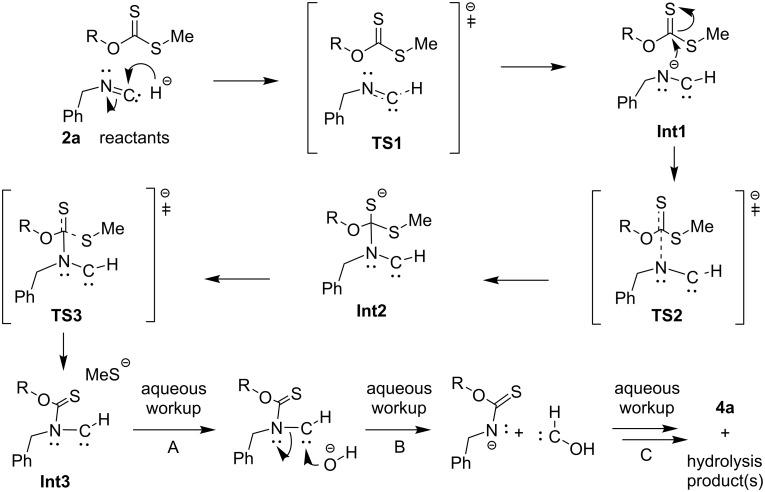
Proposed general reaction mechanism for the formation of carbamothioates (e.g., **4a**) from xanthate esters (e.g., **1a**) and benzyl isocyanides (e.g., **2a**). The counterion in all steps is presumed to be Na^+^ from NaH.

**Figure 4:**
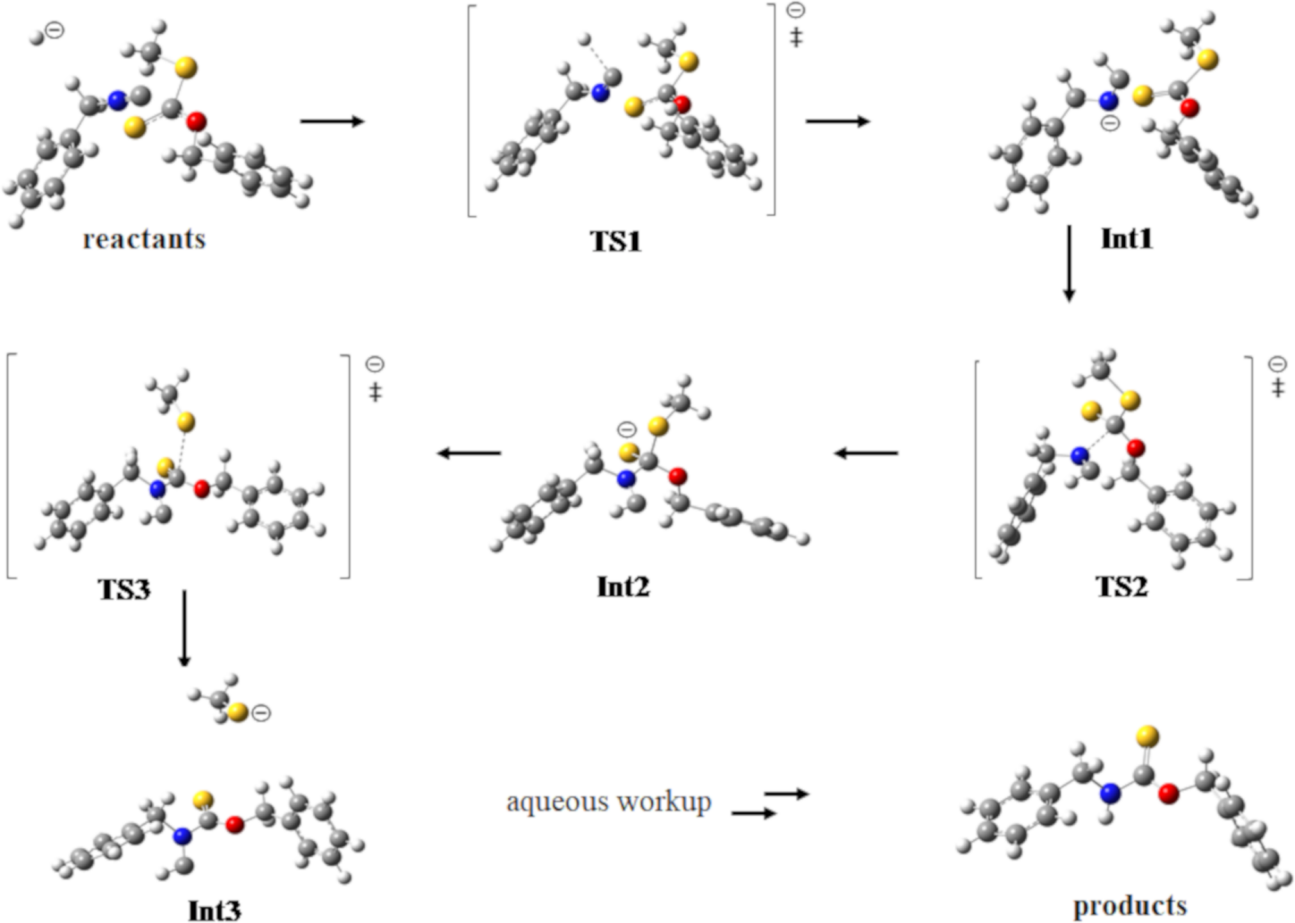
Optimized geometries of the reactants, transition states, intermediates, and products of the proposed reaction mechanism, as shown schematically in Scheme 2, determined at the B3LYP/6-311++G(d,p) level of theory in DMF (using a PCM).

**Figure 5:**
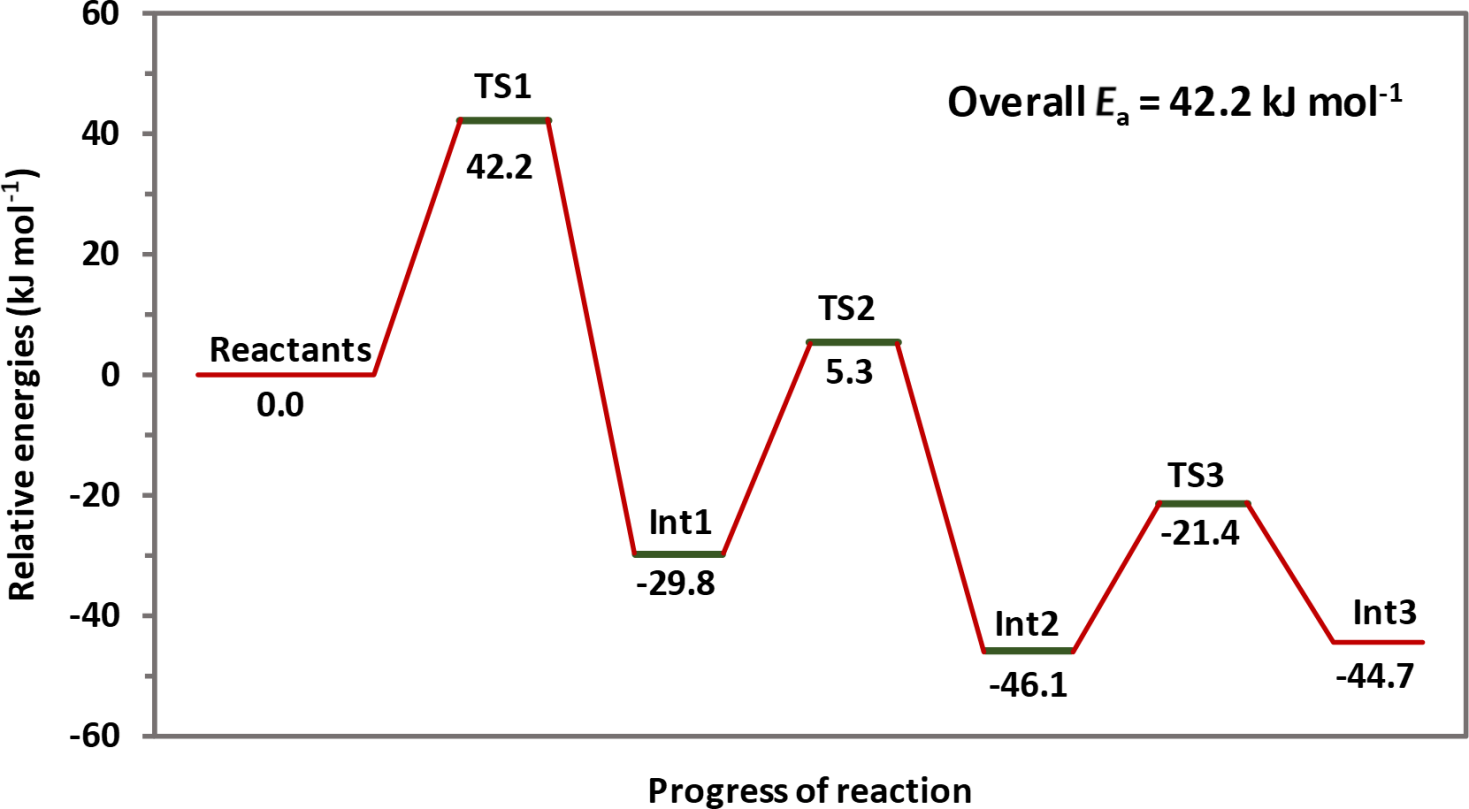
Relative energies of the reactants, transition states (**TS1**–**TS3**), and intermediates (**Int1**–**Int3**) of the proposed reaction mechanism, computed at the B3LYP/6-311++G(d,p) level of theory in DMF as the solvent.

Several possible reaction mechanisms were considered to account for the unexpected products obtained. Ultimately, we employed quantum chemical calculations to shed light on the most probable reaction pathway for the observed products, as shown in Scheme 2. For simplicity, the reaction of benzyl isocyanide (**2a**) with *O*-benzyl *S*-methyl dithiocarbonate (**1a**) was chosen for the calculations, forming the intermediates **Int1**–**3** via the most probable transition states **TS1**–**3**, respectively, which, after hydrolysis, formed the observed product **4a**. To simplify the quantum chemical calculations, the reactions shown in Scheme 2 involve a hydride as the nucleophile or base [34], although it is possible that dimethylamide, formed from the reaction of sodium hydride with DMF [35], could be the initiating nucleophile/base. All computations were carried out with Gaussian 09 [36]. The HF/6-31G(d) level of theory in the gas phase was only used to locate the transition state geometries. An intrinsic reaction coordinate (IRC) analysis was conducted for each transition state studied in this work to confirm that the transition states were associated with the respective minima. The final IRC structures were further optimized (Figure S14, Supporting Information File 1). The geometries of all reactants, transition states, and intermediates were then fully optimized at the B3LYP/6-311++G(d,p) level of theory in the DMF solvent phase using the polarized continuum model (PCM). Vibrational frequencies for all of the optimized structures were calculated to ensure the presence of a single imaginary frequency for each transition state, and the absence of imaginary frequencies for reactants, intermediates, and products and also to obtain thermal corrections for energies at 298.15 K. The optimized geometries of reactants, transition states, intermediates, and the product of the proposed reaction mechanism are shown in Figure 4. The relative energies are shown in Figure 5 and are summarized in Table S3, Supporting Information File 1. However, it should be noted that quantum chemical calculations for the hydrolysis steps subsequent to the formation of **Int3** (i.e., steps A–C leading to the hydrolysis products in Scheme 2) were not conducted.

The proposed mechanism (Scheme 2 and Figure 4) involves several steps, the most significant one being the formation of the anion **Int1** by hydride addition to the terminal carbon atom of the isocyanide group in **2a** via transition state **TS1**. This anion undergoes nucleophilic addition to the thiocarbonyl moiety of the xanthate **1a** to generate the intermediate **Int2** via transition state **TS2**. In the next step, elimination of the thiomethyl group via transition state **TS3** forms the third intermediate **Int3**, consisting of a carbene and a thiolate anion. The steps leading to the observed product carbamothioate **4a** occur from the final quenching hydrolysis of **Int3**, which occurs via several energetically favorable steps (e.g., **A**–**C**), as has been reported for other carbene hydrolyses [37–39]. As can be seen from Figure 5, the highest activation energy barrier is 42.2 kJ/mol.

We had previously considered an alternative mechanism in which a benzylic proton is instead removed by the base [40]. For the previous mechanism, which we have now recalculated at the B3LYP/6-311++G(d,p) level of theory in DMF (using a PCM, see Scheme S1 and Figures S15 and S16, Supporting Information File 1), an activation energy barrier of 20.6 kJ/mol was obtained for the formation of the resulting benzylic α-carbanion and H_2_. This benzylic α-carbanion could subsequently undergo a nucleophilic addition to the thiocarbonyl group via a transition state, which was analogous to **TS2** depicted in Scheme 2 above. However, in this step, instead of the nitrogen anion, the carbanion was the nucleophile. Subsequently, elimination of the thiomethyl or thiolate anion, either by a stepwise or by a concerted five-membered ring transition state, followed by several subsequent steps could lead to the observed products. However, the mechanism involving all of those steps required an improbably higher overall activation energy of 173.9 kJ/mol (Figure S16, Supporting Information File 1) for the products observed. This alternative mechanism is therefore unlikely, considering the mild conditions employed (35–45 °C), as compared to the proposed reaction mechanism shown in Scheme 2 and Figure 4, which had an overall activation energy of only 42.2 kJ/mol (Figure 5).

Due to the subsequent dilute aqueous/DMF quenching conditions, we were unable to detect the hydrolysis products by HRMS. Furthermore, using the same reaction conditions, which were employed to the *S*-methyl dithiocarbonates **1a**–**f**, but using *S*-ethyl or *S*-benzyl dithiocarbonates, none of the corresponding expected reaction products were obtained. As well, reductive quenching with aqueous NaCNBH_3_ failed to trap **Int3** and only afforded the same reaction products.

## Conclusion

There are several reports, which have discussed interesting reactions or reactivity of isocyanides. Among these are those which showed that the isocyanide carbon atom can act as either a nucleophile or an electrophile. To account for the reactions reported herein, the isocyanide carbon atom acted as an electrophile in the reaction with a hydride (or a dimethylamide anion stemming from DMF). A facile general protocol was described for the unexpected formation of carbamothioates **4a**–**l** by the reaction of the corresponding isocyanides **2a**–**d** with *S*-methyl xanthate esters (or *S*-methyl dithiocarbonates) **1a**–**f** in the presence of sodium hydride in DMF (method A). The short reaction time and simple work-up procedure were noteworthy features of this protocol. As well, these carbamothioates **4a**–**l** were also synthesized by the condensation of xanthate esters **1a**–**f** with benzylamines **5a**–**d** in the presence of sodium hydride in DMF (method B) for comparison. The reaction times required using method A were shorter than those required by method B. In most cases, rotamers of the final products were detected in the NMR spectra, and a representative DFT computational analysis conducted with **4c** (this compound also yielded a crystal structure) was in agreement with the ratio of the two rotamers that were observed in the corresponding NMR spectra. A mechanism was proposed that could be supported by quantum chemical calculations. Of course, other alternative mechanisms that can be envisioned include one in which the thiolate anions, which were generated, could also either a) regenerate dimethylamide anions from the surrounding DMF solvent; and/or b) possibly add to the isocyanide carbon atom to generate a nucleophilic nitrogen atom akin to the step that led to the analogous **TS1**. However, the fact that catalytic amounts of NaH were not sufficient to afford the observed product (Table 1, method A, entry 9) and that the reactions using method A required at least an equimolar amount of NaH (Table 1, method A, entry 8, cf. entry 1) but 2 equivalents in the other solvents suggested that the latter scenarios (a and b) were less likely and that the methylthiolate was perhaps countered by the sodium cation from sodium hydride. Further work on this and other isocyanide-mediated cyclization reactions are currently in progress in our laboratory.

## Supporting Information

File 1 Experimental procedures, analytical data, copies of ^1^H and ^13^C NMR spectra of all studied compounds, and computational details.

File 2 DFT output files.
