# Supplementary material for: The reaction of arylmethyl isocyanides and arylmethylamines with xanthate esters: a facile and unexpected synthesis of carbamothioates
Source: Beilstein J Org Chem. 2020 Feb 3;16:159–67. doi: 10.3762/bjoc.16.18 (PMC7034244; doi:10.3762/bjoc.16.18)
Supplement: File 1 — Experimental procedures, analytical data, copies of 1H and 13C NMR spectra of all studied compounds, and computational details. [file Beilstein_J_Org_Chem-16-159-s001.pdf]

BEILSTEIN JOURNAL OF ORGANIC CHEMISTRY

Supporting Information

for

The reaction of arylmethyl isocyanides and arylmethylamines with xanthate esters: a facile and unexpected synthesis of carbamothioates

Narasimhamurthy Rajeev, Toreshettahally R. Swaroop, Ahmad I. Alrawashdeh, Shofiur Rahman, Abdullah Alodhayb, Seegehalli M. Anil, Kuppalli R. Kiran, Chandra, Paris E. Georghiou, Kanchugarakoppal S. Rangappa
and Maralinganadoddi P. Sadashiva

Beilstein J. Org. Chem. **2020,** *16,* 159–167. doi:10.3762/bjoc.16.18

Experimental procedures, analytical data, copies of ^{1}H and ^{13}C NMR spectra of all studied compounds, and computational details

License and Terms: This is a supporting information file under the terms of the Creative Commons Attribution License (https://creativecommons.org/licenses/by/4.0). Please note that the reuse, redistribution and reproduction in particular requires that the authors and source are credited.
The license is subject to the *Beilstein Journal of Organic Chemistry* terms and conditions: (https://www.beilstein-journals.org/bjoc)

Table of Contents

	Page
Experimental Section	S2
Table 1: Crystal data and structure refinement for **4c**	S6
Table 2: Bond lengths [Å] and angles [°] for **4c**	S7
Figure S1: ^{1}H and ^{13}C NMR spectra of **4a**	S8
Figure S2: ^{1}H and ^{13}C NMR spectra of **4b**	S9
Figure S3: ^{1}H and ^{13}C NMR spectra of **4c**	S10
Figure S4: ^{1}H and ^{13}C NMR spectra of **4d**	S11
Figure S5: ^{1}H and ^{13}C NMR spectra of **4e**	S12
Figure S6: ^{1}H and ^{13}C NMR spectra of **4f**	S13
Figure S7: ^{1}H and ^{13}C NMR spectra of **4g**	S14
Figure S8: ^{1}H and ^{13}C NMR spectra of **4h**	S15
Figure S9: ^{1}H and ^{13}C NMR spectra of **4i**	S16
Figure S10: ^{1}H and ^{13}C NMR spectra of **4j**	S17
Figure S11: ^{1}H and ^{13}C NMR spectra of **4k**	S18
Figure S12: ^{1}H and ^{13}C NMR spectra of **4l**	S19
Figure S13: ^{1}H spectra of **4c** in DMSO-d_6 after heating to 60 °C	S20
Figure S14: Total energies (hartrees) along the intrinsic reaction coordinates (IRC)	S21
Table S3: Relative electronic energies (E_{ele}), thermal energies ($E_{thermal}$)	S21
Scheme S1: The alternative mechanism previously considered	S22
Figure S15: The optimized geometries	S23
Figure S16: Relative energies (E_a; kJ/mol) for the alternative mechanism	S24
References	S24

1. Experimental Section

1.1. General information. The starting materials were purchased from commercial sources. The solvents were of analytical grade and were used without further purification. Reactions were monitored by thin-layer chromatography (TLC) using precoated sheets of silica gel 60 (Merck 60 F_{254}, 0.25 mm thickness) and visualization under UV light. The melting points were determined on a SELACO melting point apparatus and are uncorrected. ^{1}H NMR (400 MHz) and ^{13}C NMR (100 MHz) spectra were obtained using an Agilent NMR spectrometer. Chemical shifts (δ) are given in parts per million (ppm) using the residue solvent ($CDCl_3$) peak as reference relative to TMS. Coupling constant (*J*) values are given in Hz. Mass spectral analysis was performed using a Waters Synapt G2 mass spectrometer. Infrared spectra were recorded on a Shimadzu FT-IR model 8300 spectrophotometer. The X-ray diffraction data were collected on a Bruker SMART APEX II X-ray diffractometer with graphite monochromated MoKα radiation. Raw data was processed and reduced by using APEX2 and SAINT. [SI1] The structure was solved by direct methods and full matrix least squares refinement was carried out using SHELXS-97/SHELXL-97, respectively [SI2]. Geometry optimization and molecular energies were calculated by using B3LYP/ 6-311 ++G(d,p) basis set [SI3].

1.2. General procedure for the synthesis of thionocarbamates:

Method A: To a suspension of sodium hydride (2.0 mmol) in DMF (2.0 mL), a mixture of xanthate ester **1** (1.0 mmol) and arylmethyl isocyanide **2** (1.0 mmol) in DMF (2.0 mL) was added at 0 °C. The stirring was continued for 15 to 30 min (monitored by TLC) at 35 to 45°C. After the completion of the reaction, the reaction mixture was quenched by adding ice cold water (25 mL), extracted with ethyl acetate (2 × 25 mL). The combined organic layer was washed with water (25 mL), brine (25 mL), dried over anhydrous sodium sulfate and concentrated under reduced pressure to get crude products, which were purified by column chromatography over silica gel using 10% ethyl acetate in hexane.

Method B: To a suspension of sodium hydride (2.0 mmol) in DMF (2.0 mL) at 0 °C, a mixture of xanthate ester **1** (1.0 mmol) and arylmethylamine **5** (1.0 mmol) in DMF (2.0 mL) was added and stirred for 1 to 1.5 h (monitored by TLC) at 35 to 45°C. After the completion of reaction, the reaction mixture was quenched by adding ice cold water (25 mL), extracted with ethyl acetate (2 × 25 mL). The combined organic layer was washed with water (25 mL), brine (25 mL), dried over anhydrous sodium sulphate and concentrated under

reduced pressure to get crude products, which were purified by column chromatography over silica gel using 10% ethyl acetate in hexane. The yields from Method B are shown in parentheses.

O-benzyl benzylcarbamothioate **(4a)**. Colourless solid (mp 52-54 °C). 85%, 220 mg (80%, 210 mg). Rotamers ratio 66:34. IR (KBr, cm^{-1}) ν 636, 1069, 1285, 1328, 2936, 3256. ^{1}H NMR ($CDCl_3$, 400 MHz, δ) 4.44 (d, *J* = 6.0 Hz, 2H, CH_2), 4.76 (d, *J* = 5.6 Hz, 2H, CH_2), 5.51 (s, 2H, CH_2), 5.55 (s, 2H, CH_2), 6.58 (s, 1H, NH), 7.0 (s, 1H, NH), 7.19-7.39 (m, 20H, Ar-H). ^{13}C NMR ($CDCl_3$, 100 MHz, δ) 190.3, 189.7, 136.7, 135.7, 128.8, 128.8, 128.7, 128.5, 128.5, 128.4, 128.3, 128.2, 127.9, 127.9, 127.7, 127.6, 127.2, 73.3, 72.1, 49.4, 47.3 (1 peak lost). HRMS (ESI) $[M+H]^+$ Anal. Calcd for $C_{15}H_{16}NOS$: 258.0953; Found: 258.0957.

O-benzyl (4-methylbenzyl)carbamothioate **(4b)**. Colourless solid (mp 60-62°C). 84%, 230 mg (82%, 220 mg). Rotamers ratio 64:36. IR (KBr, cm^{-1}) ν 626, 1055, 1274, 1326, 2920, 3197. ^{1}H NMR ($CDCl_3$, 400 MHz, δ) 2.33 (s, 6H, $2CH_3$), 4.42 (d, *J* = 5.2 Hz, 2H, CH_2), 4.71 (d, *J* = 5.2 Hz, 2H, CH_2), 5.49 (s, 2H, CH_2), 5.55 (s, 2H, CH_2), 7.11-7.37 (m, 18H, Ar-H). ^{13}C NMR ($CDCl_3$, 100 MHz, δ) 190.0, 189.3, 137.4, 129.4, 129.4, 128.8, 128.5, 128.5, 128.3, 128.3, 128.1, 127.9, 127.7, 127.1, 72.0, 71.1, 47.2, 45.2, 29.6, 21.0 (4 peaks lost). HRMS (ESI) $[M+H]^+$ Anal. Calcd for $C_{16}H_{18}NOS$: 272.1109; Found: 272.1104.

O-benzyl (4-fluorobenzyl)carbamothioate **(4c)**. Yellow solid (mp 72-75°C). 87%, 240 mg (77%, 210 mg). Rotamers ratio 65:35. IR (KBr, cm^{-1}) ν 634, 1056, 1265, 1326, 2920, 3256. ^{1}H NMR ($CDCl_3$, 400 MHz, δ) 4.42 (d, *J* = 6.0 Hz, 2H, CH_2), 4.73 (d, *J* = 5.6 Hz, 2H, CH_2), 5.49 (s, 2H, CH_2), 5.53(s, 2H, CH_2), 6.52 (s, 1H, NH), 6.96 (s, 1H, NH), 6.97-7.05 (m, 4H, Ar-H), 7.14-7.18 (t, *J* = 5.2Hz, 1H, ArH), 7.21-7.25 (m, 1H, Ar-H), 7.27-7.38 (m, 12H, Ar-H). ^{13}C NMR ($CDCl_3$, 100 MHz, δ) 190.4, 189.6, 163.6 (*J*=34.0 Hz), 161.1 (*J*=34.0 Hz), 135.6, 132.5, 129.7, 129.6, 129.4, 129.3, 128.8, 128.6, 128.4, 128.3, 127.2, 115.7, 115.5, 115.4, 73.4, 72.2, 48.6, 46.6. HRMS (ESI) $[M+H]^+$ Anal. Calcd for $C_{15}H_{15}FNOS$: 276.0858; Found: 276.0851.

O-(2-methylbenzyl) benzylcarbamothioate **(4d)**. Viscous liquid. 81%, 220 mg (81%, 220 mg). Rotamers ratio 66:34. IR (KBr, cm^{-1}) ν 696, 1056, 1323, 1596, 2865, 3206. ^{1}H NMR ($CDCl_3$, 400 MHz, δ) 2.28 (s, 3H, Ar-CH_3), 2.36 (s, 3H, Ar-CH_3), 4.41 (d, *J* = 6.4 Hz, 2H, Ar-CH_2), 4.76 (d, *J* = 5.6 Hz, 2H, Ar-CH_2), 5.50-5.53 (two singlet, 4H, $2CH_2$), 7.15-7.36 (m, 18H, Ar-H). ^{13}C NMR ($CDCl_3$, 100 MHz, δ) 190.9, 190.2, 138.9, 138.6, 136.9, 136.7, 136.9, 133.9, 133.5, 130.3, 130.3, 129.6, 129.5, 128.7, 128.0, 127.9, 127.6, 126.0, 125.9, 72.0, 70.7, 49.2, 47.3, 18.9, 18.8 (3 peaks lost). HRMS (ESI) $[M+H]^+$ Anal. Calcd for $C_{16}H_{18}NOS$: 272.1109; Found: 272.1105.

O-(3-methoxybenzyl) (4-fluorobenzyl)carbamothioate **(4e)**. Colourless solid (mp 74-76°C). 83%, 250 mg (75%, 230 mg). Rotamers ratio 100:0. IR (KBr, cm^{-1}) ν 690, 1097, 1353, 1482, 1584, 2852, 3015. ^{1}H NMR ($CDCl_3$, 400 MHz, δ) 3.77 (s, 3H, OCH_3), 4.15 (s, 2H, CH_2), 4.43 (d, *J* = 5.6 Hz, 2H, CH_2), 5.61 (s, 1H, NH), 6.78 (dd, *J* = 8.2 Hz, 2.4 Hz, 1H, Ar-H), 6.88-6.92 (m, 2H, Ar-H), 7.00 (t, *J* = 8.4 Hz, 2H, Ar-H), 7.18-7.25 (m, 3H, Ar-H). ^{13}C NMR ($CDCl_3$, 100 MHz, δ) 190.1, 163.5 (*J*=31.0 Hz), 159.7, 139.6, 129.5, 129.4, 121.1, 115.6, 115.5, 114.3, 112.9, 55.1, 44.7, 34.3. HRMS (ESI) $[M+H]^+$ Anal. Calcd for $C_{16}H_{17}FNO_2S$: 306.0964; Found: 306.0969.

O-(3-methoxybenzyl) (4-chlorobenzyl)carbamothioate **(4f)**. Viscous liquid. 79%, 250 mg (83%, 270 mg). Rotamers ratio 100:0. IR (KBr, cm^{-1}) ν 692, 1040, 1318, 1486, 1593, 2920, 3025. ^{1}H NMR ($CDCl_3$, 400 MHz, δ) 3.80 (s, 3H, OCH_3), 4.15 (s, 2H, CH_2), 4.43 (d, *J* = 5.6 Hz, 2H, CH_2), 5.64 (s, 1H, NH), 6.76-6.79 (m, 1H, Ar-H), 6.89-7.17 (m, 2H, Ar-H), 7.18-7.29 (m, 5H, Ar-H). ^{13}C NMR ($CDCl_3$, 100M Hz, δ) 190.1, 159.7, 138.3, 137.3, 136.3, 129.5, 128.9, 128.8, 121.0, 114.2, 112.9, 55.1, 34.3, 29.6. HRMS (ESI) $[M+H]^+$ Anal. Calcd for $C_{16}H_{17}ClNO_2S$: 322.0669; Found: 322.0661.

O-(4-bromobenzyl) benzylcarbamothioate **(4g)**. Yellow solid (mp 110-115°C). 80%, 270 mg (82%, 270 mg). Rotamers ratio 56:44. IR (KBr, cm^{-1}) ν 746, 1041,1294, 1593, 2920, 3204. ^{1}H NMR ($CDCl_3$, 400 MHz, δ) 4.43 (d, *J* = 6.0 Hz, 2H, CH_2), 4.75 (d, *J* = 5.6 Hz, 2H, CH_2), 5.45-5.48 (two singlet, 4H, $2CH_2$), 6.6 (s, 1H, NH), 7.00 (s, 1H, NH), 7.16-7.34 (m, 16H, Ar-H), 7.35-7.48 (m, 2H, Ar-H). ^{13}C NMR ($CDCl_3$, 100 MHz, δ) 190.5, 190.0, 136.5, 134.8, 131.6, 131.6, 130.6, 130.5, 130.0, 129.8, 128.8, 128.8, 128.0, 128.0, 127.5, 72.3, 71.0, 49.5, 47.4 (3 peaks lost). HRMS (ESI) $[M+H]^+$ Anal. Calcd for $C_{15}H_{15}BrNOS$: 336.0058 and 338.0037; Found: 336.0051 and 338.0032.

O-(4-bromobenzyl) (4-methylbenzyl)carbamothioate **(4h)**. Yellow solid (mp 96-98 °C). 76%, 260 mg (86%, 300 mg). Rotamers ratio 58:42. IR (KBr, cm^{-1}) ν 747, 1061, 1285, 1549, 2923, 3225. ^{1}H NMR ($CDCl_3$, 400 MHz, δ) 2.31 & 2.33 (two singlet, 6H, $2CH_3$), 4.39 (d, *J* = 5.6 Hz, 2H, CH_2), 4.70 (d, *J* = 5.2 Hz, 2H, CH_2), 5.44 & 5.49 (s, 2H, CH_2), 6.5 (s, 1H, NH), 6.9 (s, 1H, NH), 7.07-7.25 (m, 14H, Ar-H), 7.39-7.48 (m, 2H, Ar-H). ^{13}C NMR ($CDCl_3$, 100 MHz, δ) 190.7, 189.8, 137.7, 134.8, 133.5, 131.6, 131.6, 130.5, 129.9, 129.8, 129.4, 129.2, 128.0, 127.9, 127.5, 122.3, 72.3, 70.9, 49.3, 47.1, 29.6, 21.0 (2 peaks lost). HRMS (ESI) $[M+H]^+$ Anal. Calcd for $C_{16}H_{17}BrNOS$: 350.0214 and 352.0194; Found: 350.0219 and 352.0198.

O-butyl (4-fluorobenzyl)carbamothioate **(4i)**. Viscous liquid. 86%, 210 mg (74%, 180 mg). Rotamers ratio 60:40. IR (KBr, cm^{-1}) ν 626, 1057, 1323, 1584, 1695, 3076, 3235. ^1H NMR ($CDCl_3$, 400 MHz, δ) 0.83-0.94 (m, 6H, $2CH_3$), 1.24-1.43 (m, 4H, $2CH_2$), 1.62-1.71 (m, 4H, $2CH_2$), 4.49-4.69 (m, 4H, $2CH_2$), 4.71 (d, J = 6.0 Hz, 4H, $2CH_2$), 6.43 (s, 1H, NH), 6.98-7.03 (m, 4H, Ar-H), 7.19-7.30 (m, 4H, Ar-H). ^{13}C NMR ($CDCl_3$, 100 MHz, δ) 190.9, 190.1, 163.6 (J=28.0 Hz), 161.1 (J=28.0 Hz), 129.7, 129.6, 129.3, 129.2, 115.8, 115.7, 115.6, 115.5, 72.0, 70.6, 48.4, 46.4, 30.7, 30.6, 19.0, 13.7, 13.6. HRMS (ESI) $[M+H]^+$ Anal. Calcd for $C_{12}H_{17}FNOS$: 242.1015; Found: 242.1019.

O-butyl (4-chlorobenzyl)carbamothioate **(4j)**. Viscous liquid. 84%, 220 mg (79%, 200 mg). Rotamers ratio 62:38. IR (KBr, cm^{-1}) ν 632, 1054, 1307, 1584, 3074, 3224. ^1H NMR ($CDCl_3$, 400 MHz, δ) 0.89-1.24 (m, 6H, $2CH_3$), 1.27-1.41 (m, 4H, $2CH_2$), 1.56-1.70 (m, 4H, $2CH_2$), 4.38-4.71 (m, 4H, $2CH_2$), 4.72 (d, J = 5.6 Hz, 2H, CH_2), 6.4 (s, 1H, NH), 6.8 (s, 1H, NH),7.15-7.31 (m, 8H, Ar-H). ^{13}C NMR ($CDCl_3$, 100 MHz, δ) 190.8, 190.0, 137.2, 135.4, 129.1, 128.9, 128.8, 128.7, 127.9, 127.1, 72.1, 70.7, 48.3, 48.4, 30.6, 30.5, 29.6, 19.0, 13.7, 13.6 (2 peaks lost). HRMS (ESI) $[M+H]^+$ Anal. Calcd for $C_{12}H_{17}ClNOS$: 258.0719; Found: 258.0711.

O-(3-methylcyclohexyl) benzylcarbamothioate **(4k)**. Viscous liquid. 82%, 220 mg (81%, 210 mg). Rotamers ratio 57:43. IR (KBr, cm^{-1}) ν 626, 1318, 1236, 1523, 2896, 3221. ^1H NMR ($CDCl_3$, 400 MHz, δ) 0.79-1.05 (m, 12H, 2CH, $2CH_2$, $2CH_3$), 1.17-1.63 (m, 4H, $2CH_2$), 1.75-1.78 (m, 1H, CH), 2.09-2.12 (m, 4H, $2CH_2$), 4.4 (d, J = 6.0 Hz, 2H, CH_2), 4.73 (d, J = 5.60 Hz, 2H, CH_2), 5.23-5.29 (m, 2H, CH), 6.37 (s, 1H, NH), 7.0 (s, 1H, NH), 7.21-7.34 (m, 10H, Ar-H). ^{13}C NMR ($CDCl_3$, 100 MHz, δ) 190.2, 189.5, 136.9, 128.7, 128.7, 127.9, 127.8, 127.7, 127.6, 81.2, 79.3, 49.1, 47.0, 40.4, 40.2, 34.0, 33.9, 31.4, 31.3, 31.2, 31.2, 23.8, 23.7, 22.2, 22.2 (1 peak lost). HRMS (ESI) $[M+H]^+$ Anal. Calcd for $C_{15}H_{22}NOS$: 264.1422; Found: 264.1429.

O-(3-methylcyclohexyl) (4-fluorobenzyl)carbamothioate **(4l)**. Viscous liquid. 88%, 250 mg (71%, 200 mg). Rotamers ratio 58:42. IR (KBr, cm^{-1}) ν 638, 1222,1391, 1508, 2931, 3234. ^1H NMR ($CDCl_3$, 400 MHz, δ) 0.76-1.03 (m, 10H, $2CH_2$, $2CH_3$), 1.16-1.78 (m, 6H, $3CH_2$), 1.89-1.96 (m, 1H, CH), 2.08-2.15 (m, 2H, CH_2), 4.35 (d, J = 6.0 Hz, 1H, CH), 4.69 (d, 4H, J = 5.24 Hz, $2CH_2$), 5.20-5.29 (m, 2H, 2CH), 5.61 (s, 1H, NH), 6.37 (s, 1H, NH), 6.98-7.04 (m, 4H, Ar-H), 7.18-7.21 (m, 2H, Ar-H), 7.22-7.31 (m, 2H, Ar-H). ^{13}C NMR ($CDCl_3$, 100 MHz, δ) 189.9, 189.1, 132.8 (J=29.0 Hz), 129.7 (J=29.0 Hz), 129.6, 129.4, 129.3, 128.9, 115.7, 115.5, 81.3, 79.5, 77.3, 77.0, 76.7, 76.6, 48.3, 46.4, 40.4, 40.3, 33.9, 33.8, 31.4, 31.4, 31.3, 31.2, 31.2, 23.8, 23.7, 22.2, 22.2. HRMS (ESI) $[M+H]^+$ Anal. Calcd for $C_{15}H_{21}FNOS$: 282.1328; Found: 282.1335.

Table S1: Crystal data and structure refinement for **4c**.

Empirical formula	$C_{15}H_{14}FNOS$
Formula weight	275.33
Temperature	293 K
Wavelength	0.71073 Å
Crystal system, space group	Tryclinic, *P-1*
Unit cell dimensions	a = 5.9287 (4) Å, b = 7.3176 (4) Å, c = 16.1549 (12) Å.<br>α = 101.927 (6)°, β = 96.052 (6)º, γ = 90.294 (5)°
Volume	681.65 (8) $Å^3$
Z, Calculated density	2, 681.65 (8) Mg/m^3
F_{000}	288
Crystal size	0.30 x 0.25 x 0.20 mm
Theta range for data collection	2.6 to 27.5°.
Limiting indices	-7≤ h≤ 6, -9≤ k≤ 8, -20≤ l≤ 20
Reflections collected / unique	5575 / 2265 [R(int) = 0.025]
Refinement method	Full-matrix least-squares on F^2
Data / restraints / parameters	3125 / 0 / 172
Goodness-of-fit on F^2	1.03
Final R indices [I>2sigma(I)]	R1 = 0.0470, wR2 = 0.0991
R indices (all data)	R1 = 0.0668, wR2 = 0.1125
Largest diff. peak and hole	0.20 and -0.21 e.$Å^{-3}$

Table S2: Bond lengths [Å] and angles [°] for **4c**.

atoms	length	atoms	length
S10—C9	1.6749 (17)	C7—O8—C9	120.89 (14)
F19—C16	1.363 (2)	C9—N11—C12	124.84 (16)
O8—C7	1.449 (3)	C2—C1—C6	120.41 (18)
O8—C9	1.328 (2)	C1—C2—C3	120.2 (2)
N11—C9	1.326 (2)	C2—C3—C4	120.1 (2)
N11—C12)	1.459 (2	C3—C4—C5	119.4 (2)
C1—C2	1.376 (3)	C4—C5—C6	120.92 (19)
C1—C6	1.384 (3)	C1—C6—C5	119.08 (18)
C2—C3	1.377 (4)	C1—C6—C7	121.10 (17)
C3—C4	1.384 (4)	C5—C6—C7	119.80 (18)
C4—C5	1.382 (3)	O8—C7—C6	108.92 (15)
C5—C6	1.379 (3)	S10—C9—O8	125.28 (12)
C6—C7	1.499 (3)	S10—C9—N11	123.35 (14)
C12—C13	1.510 (2)	O8—C9—N11	111.37 (15)
C13—C18	1.388 (3)	N11—C12—C13	113.03 (14)
C13—C14	1.383 (3)	C14—C13—C18	118.53 (16)
C14—C15	1.374 (3)	C12—C13—C14	119.92 (16)
C15—C16	1.361 (3)	C12—C13—C18	121.55 (17)
C16—C17	1.368 (3)	F19—C16—C17	118.4 (2)
C17—C18	1.377 (3)	F19—C16—C15	118.77 (19)

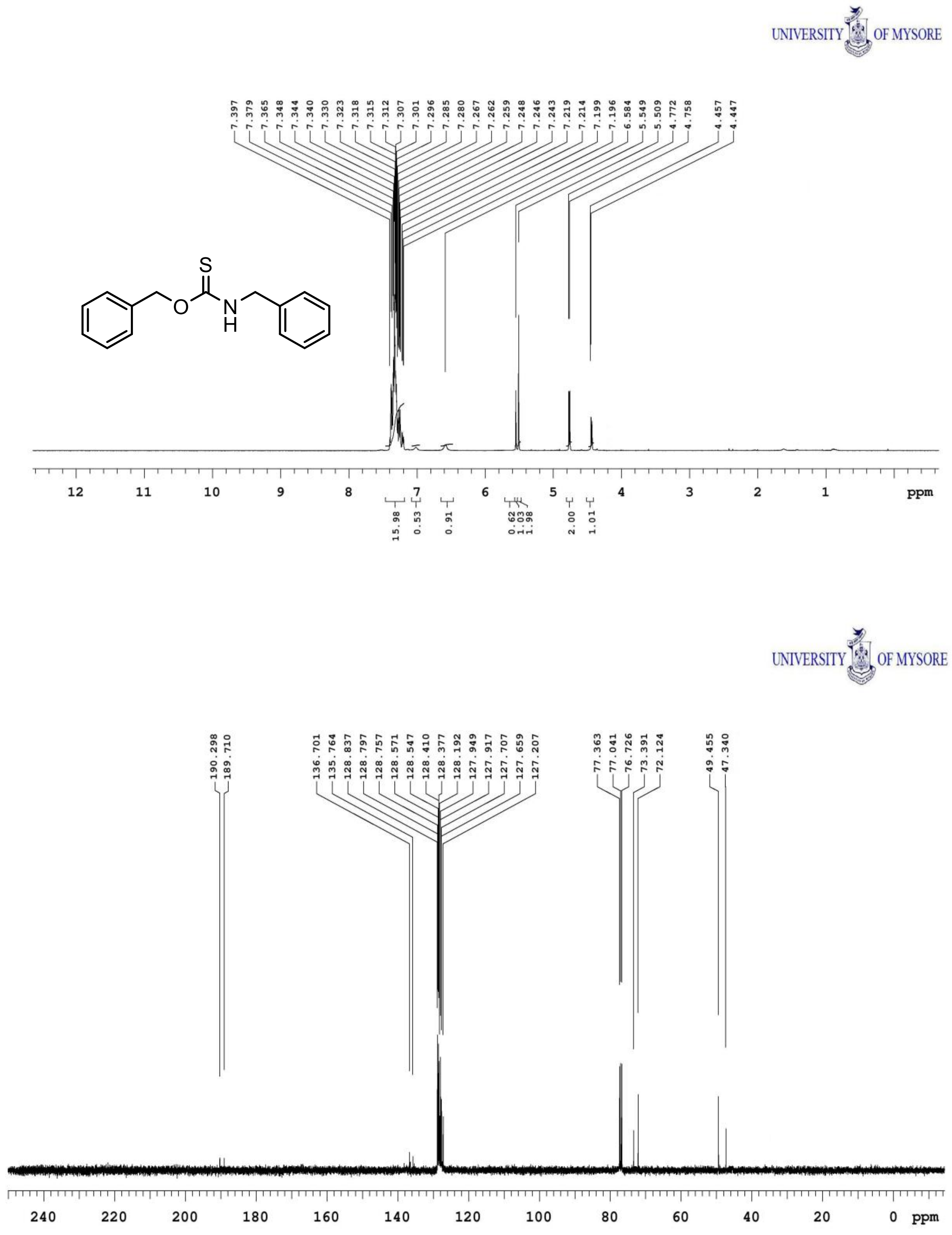

Figure S1: ^{1}H and ^{13}C NMR spectra of **4a**.

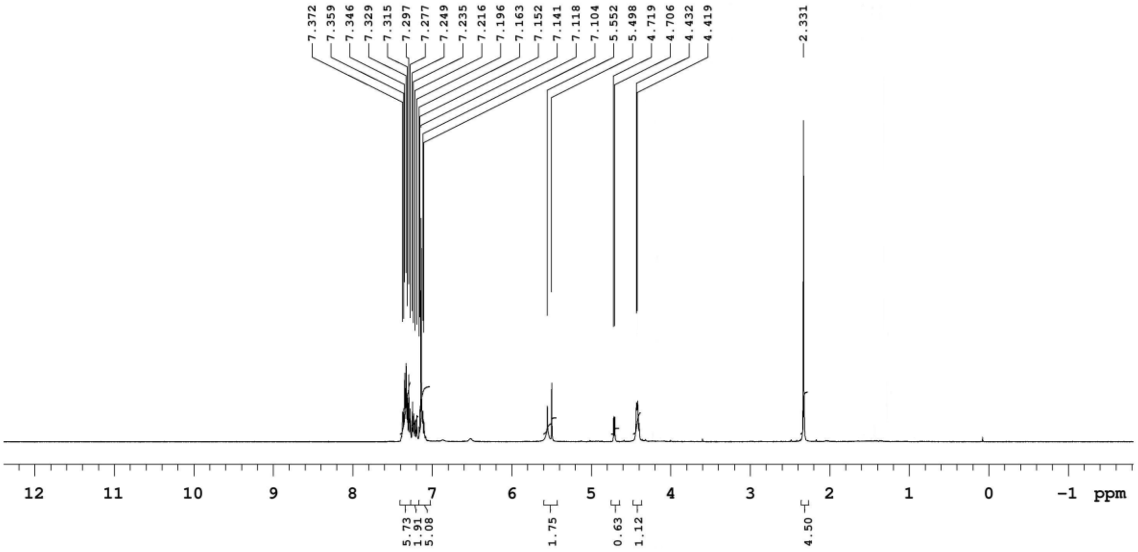

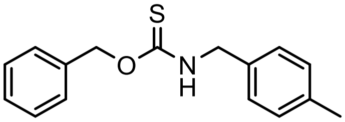

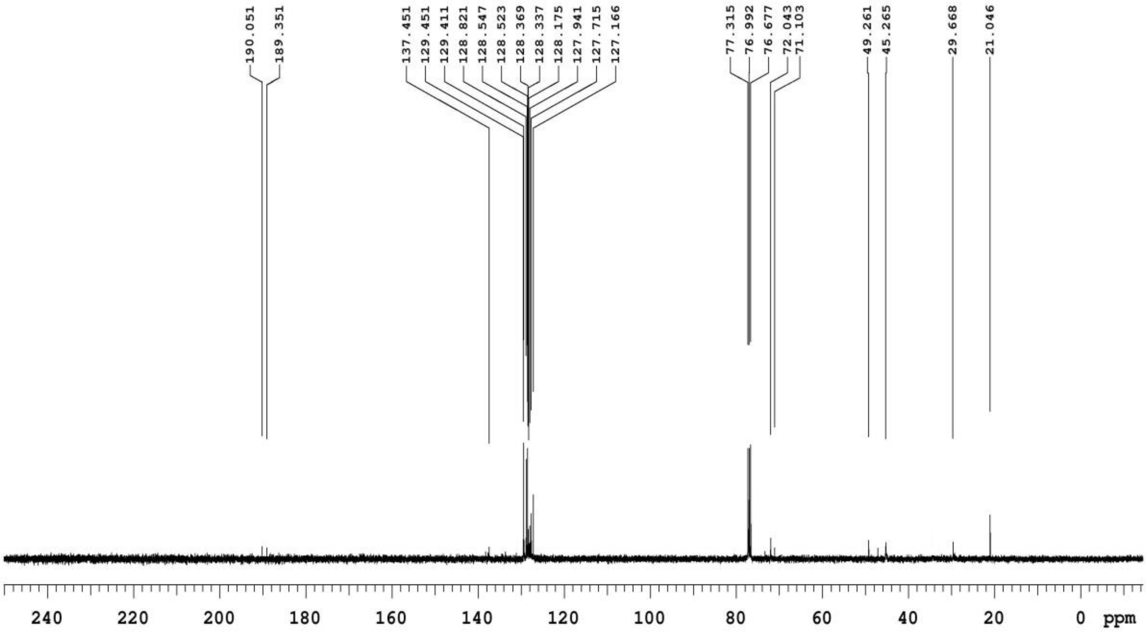

Figure S2: ^{1}H and ^{13}C NMR spectra of **4b.**

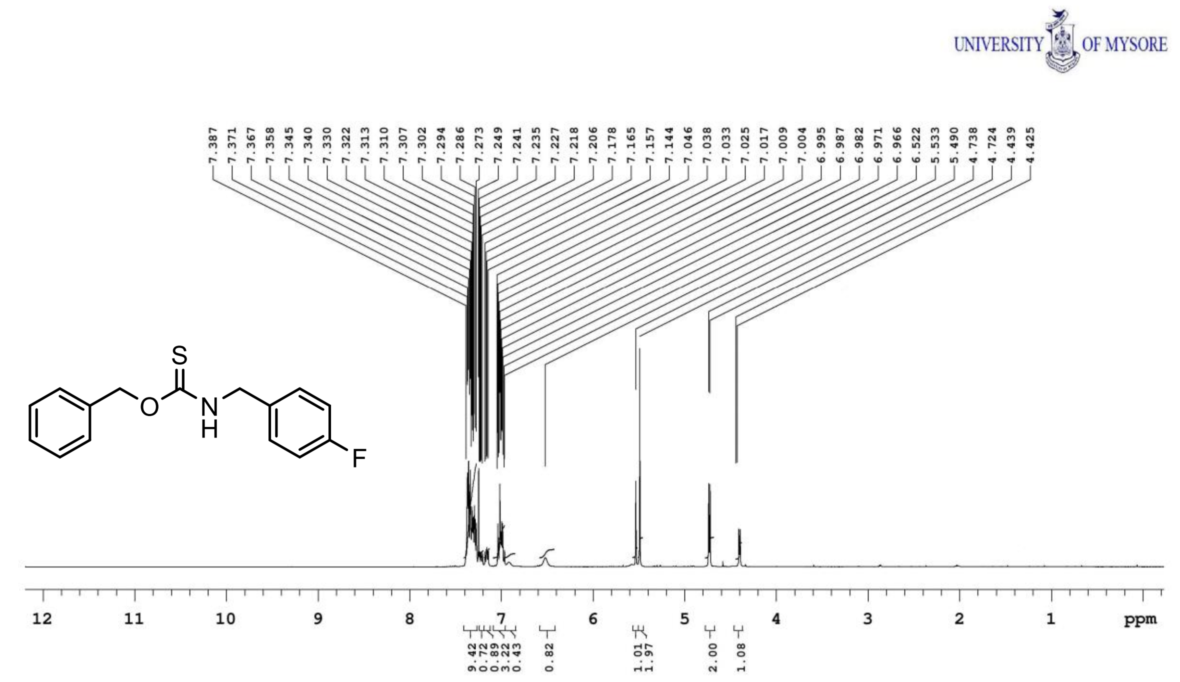

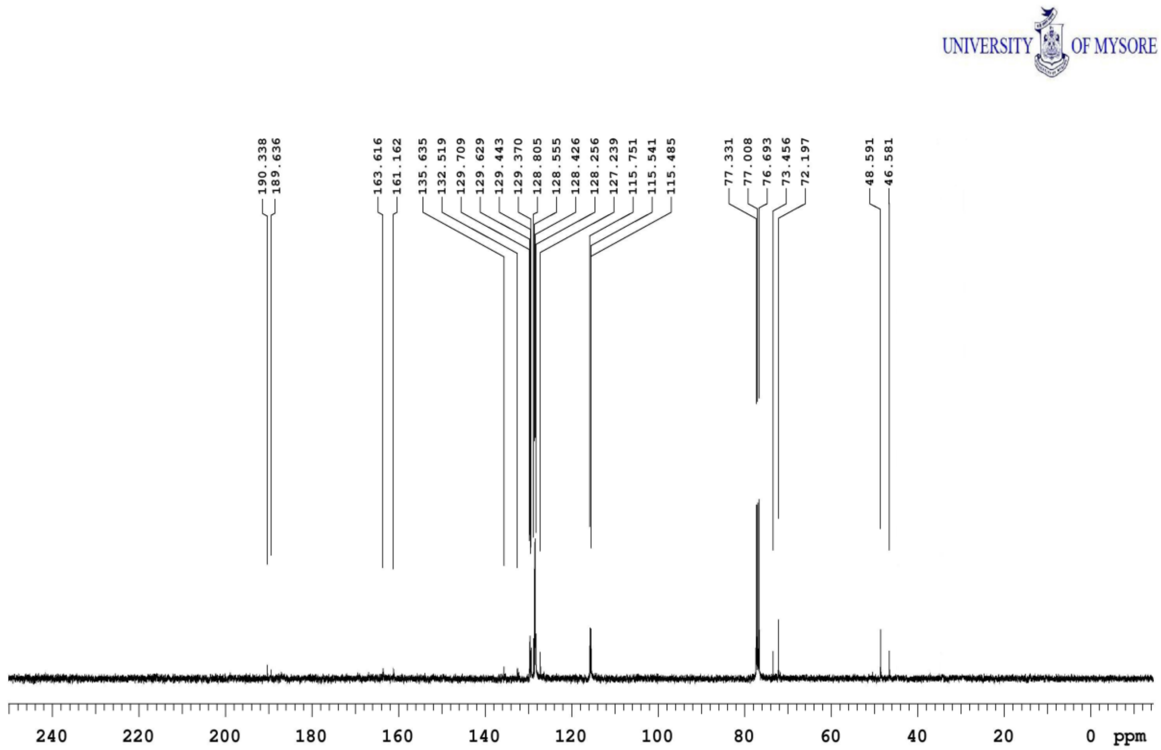

Figure S3: ^{1}H and ^{13}C NMR spectra of **4c**.

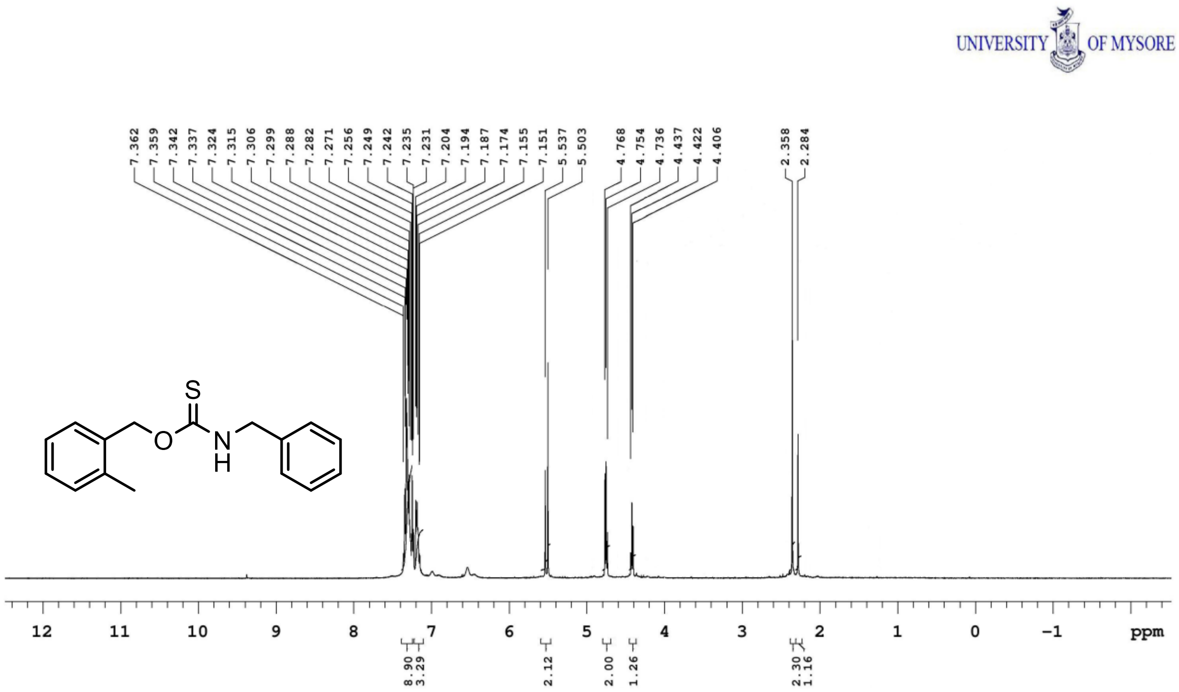

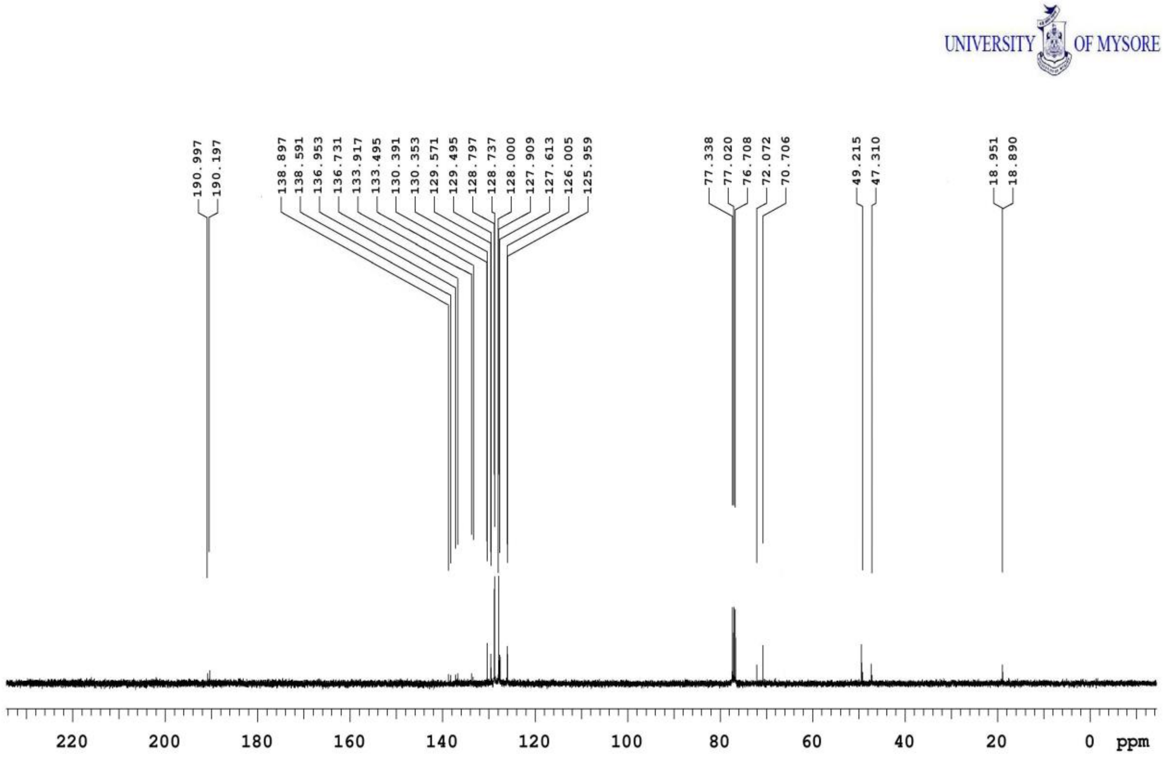

Figure S4: ^{1}H and ^{13}C NMR spectra of **4d**.

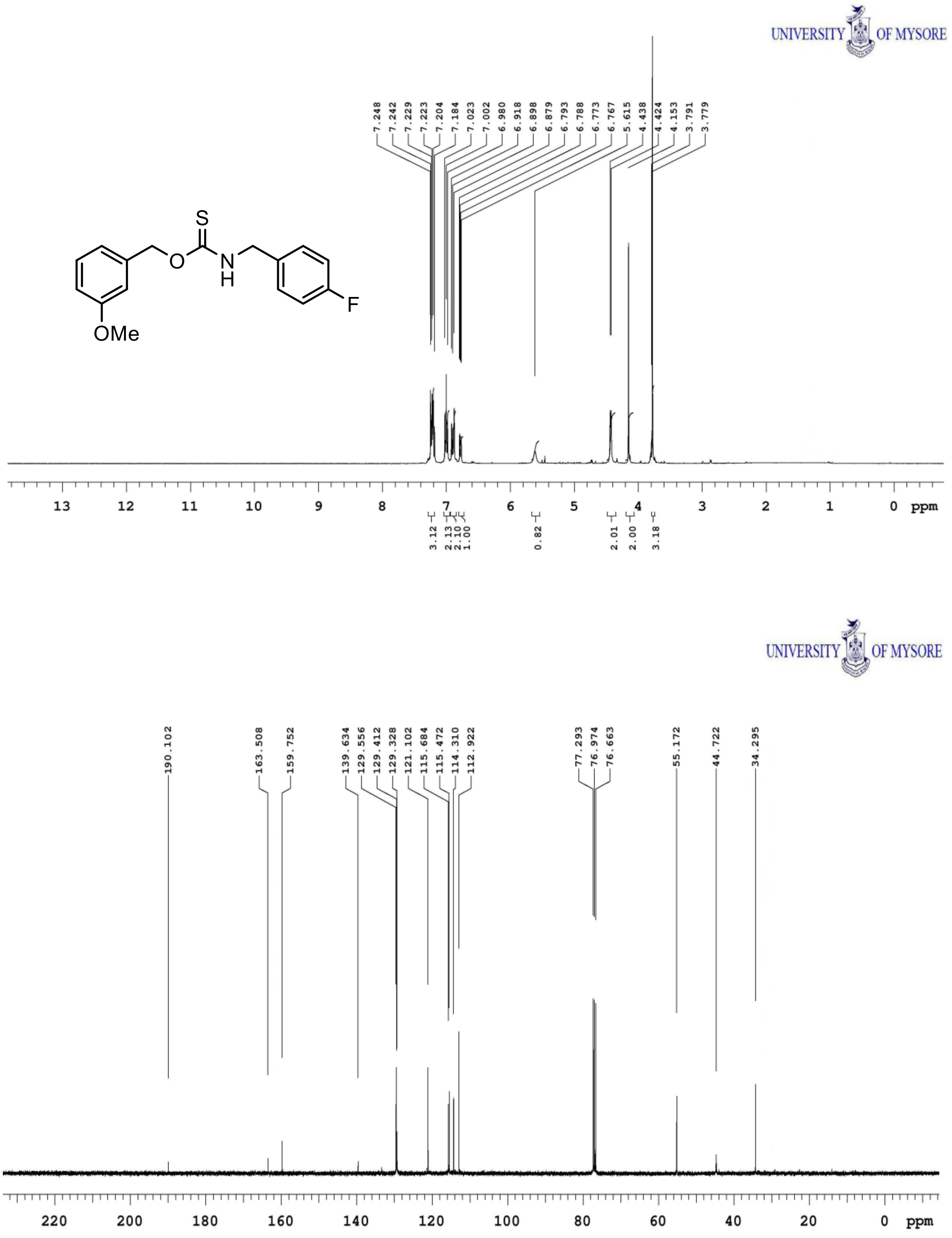

Figure S5: ^{1}H and ^{13}C NMR spectra of **4e.**

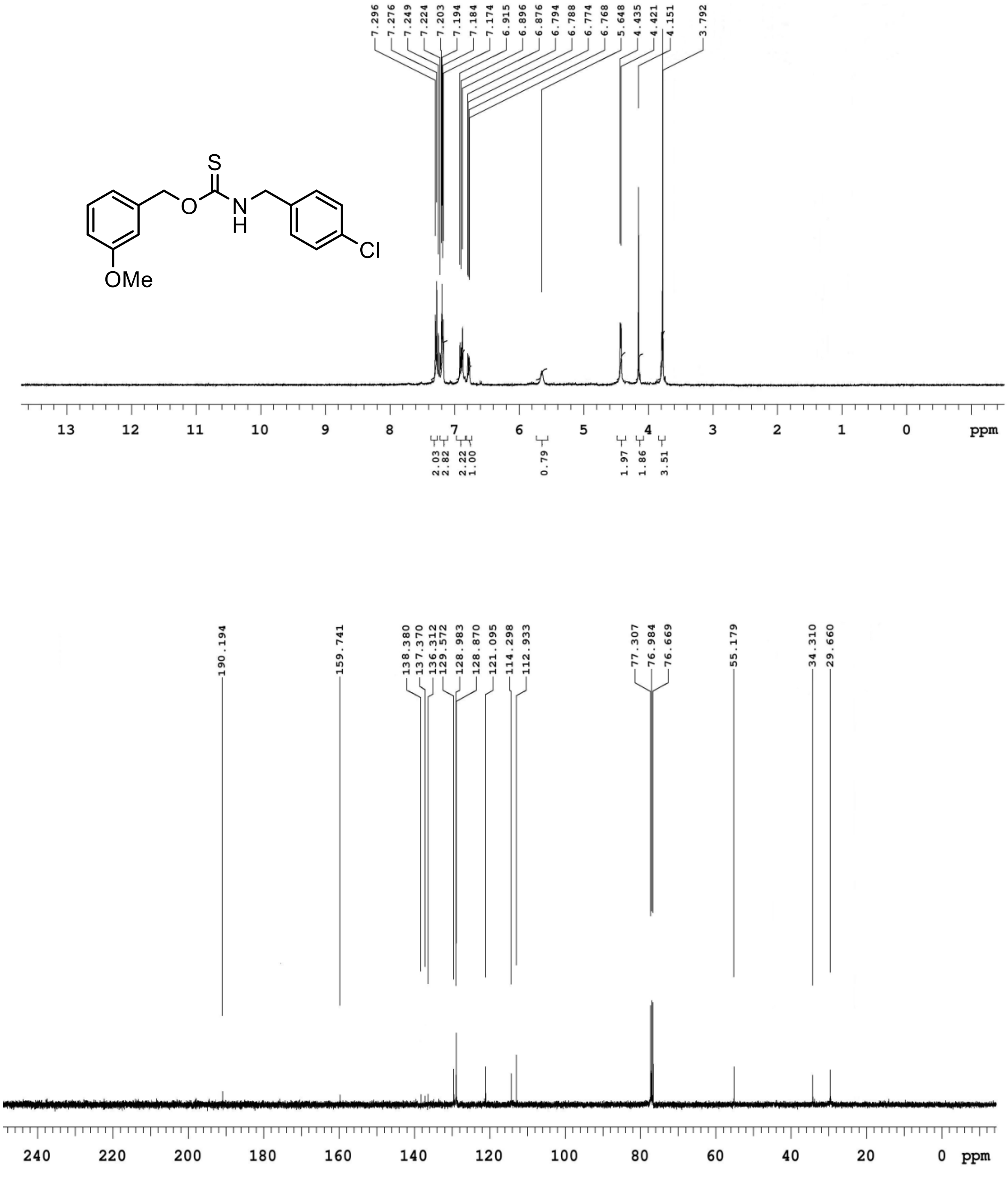

Figure S6: ^{1}H and ^{13}C NMR spectra of **4f**.

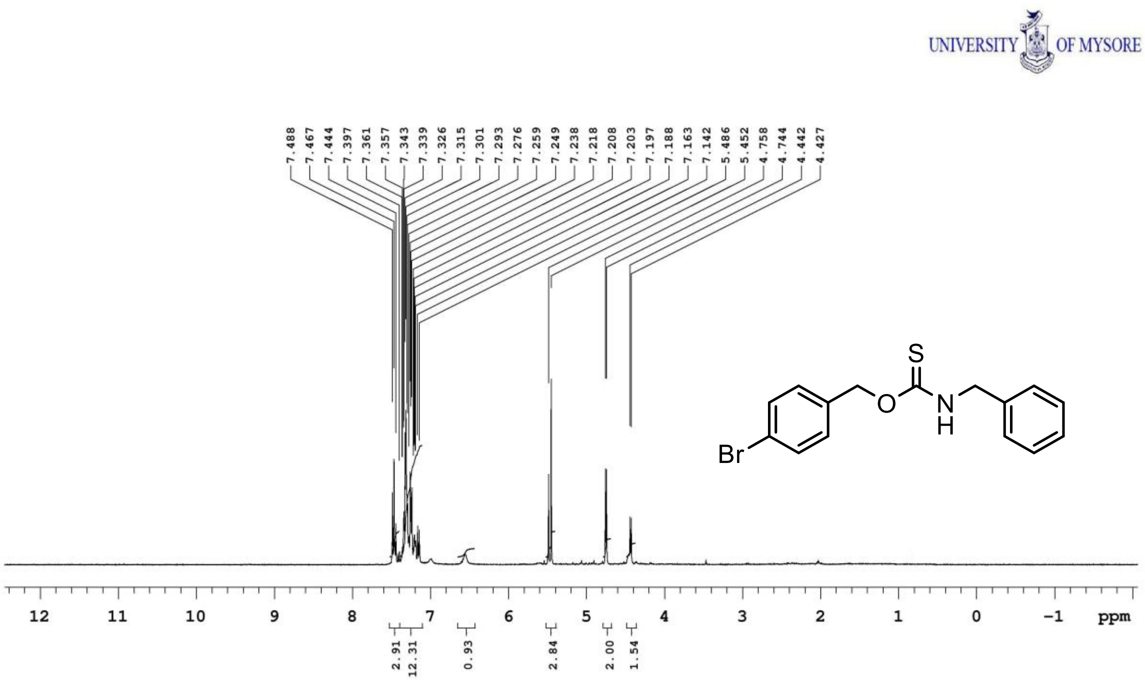

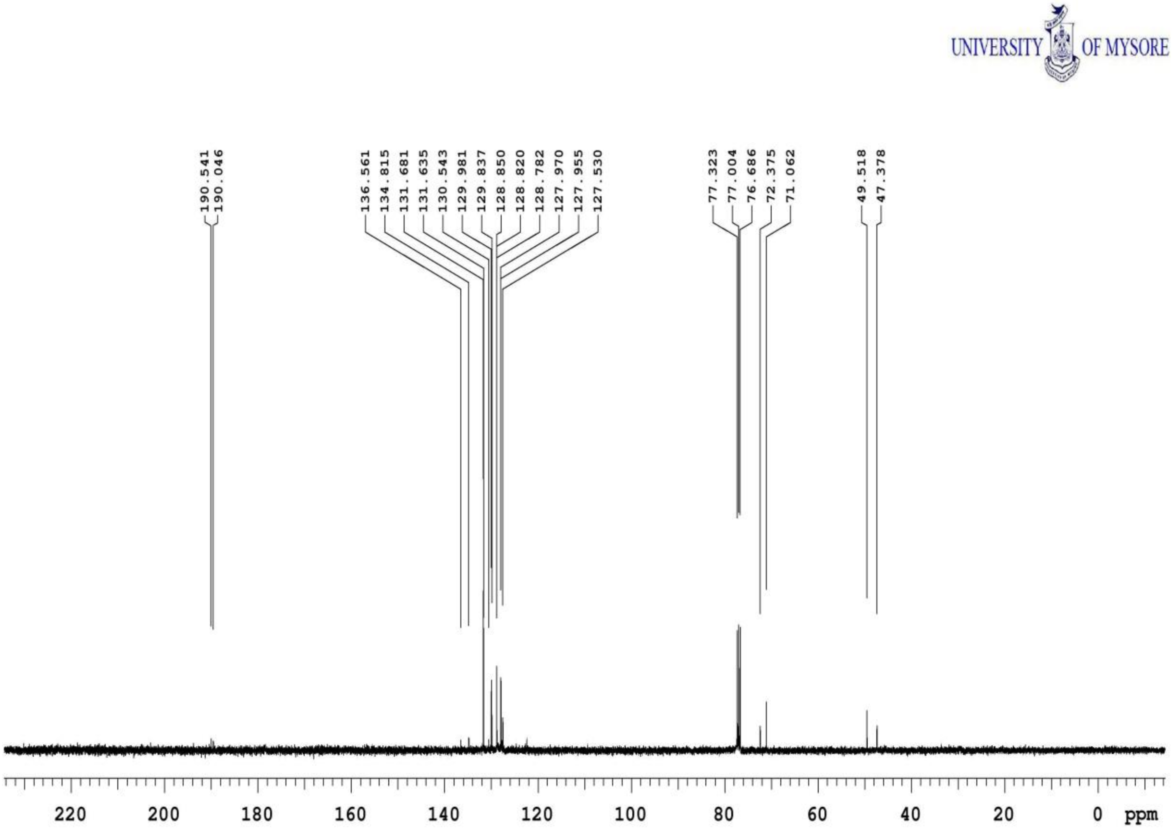

Figure S7: ^{1}H and ^{13}C NMR spectra of **4g**.

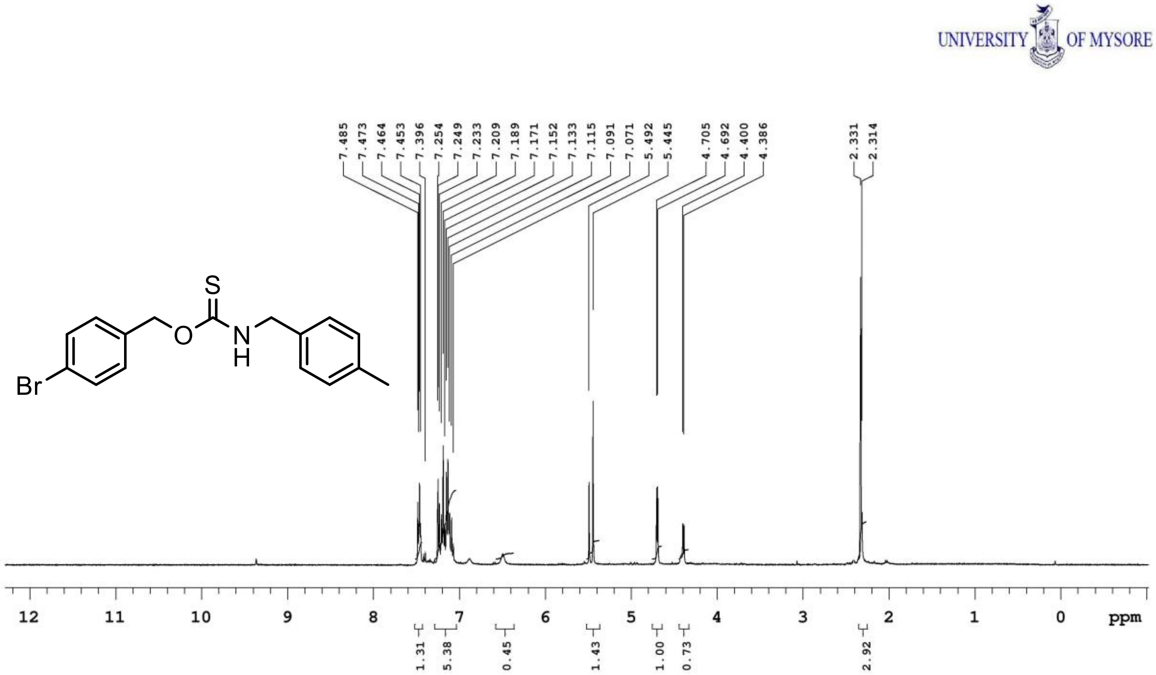

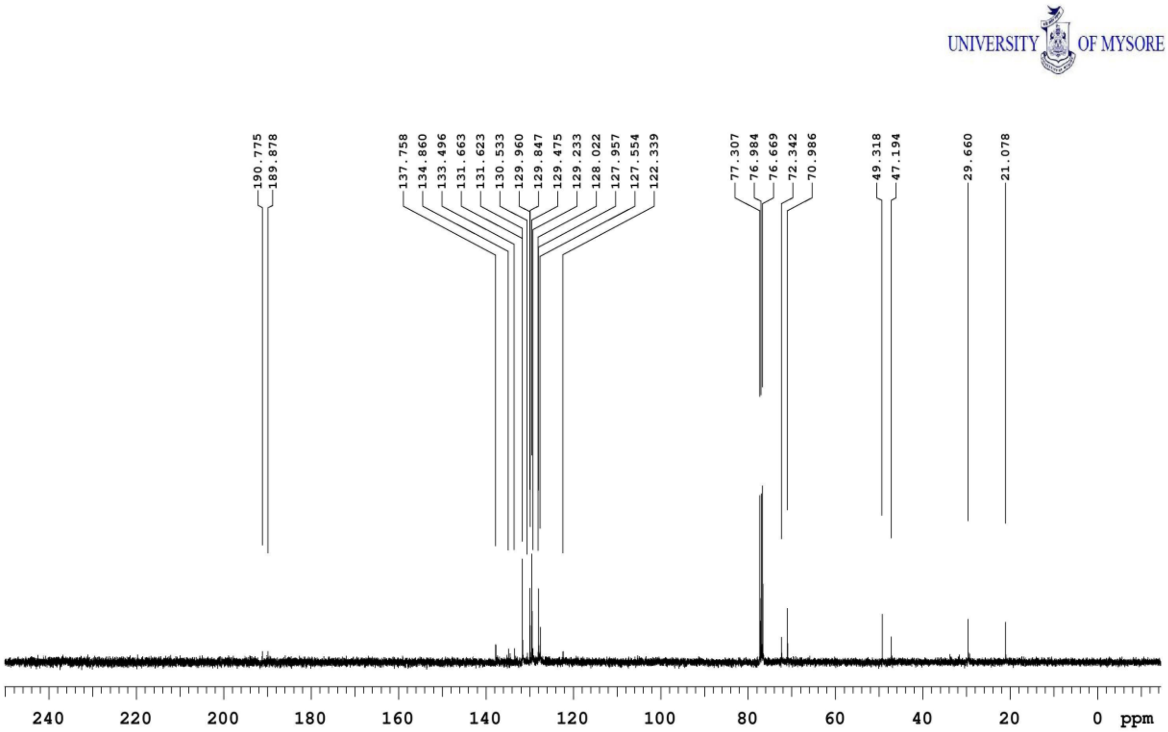

Figure S8: **^{1}**H and ^{13}C NMR spectra of **4h**.

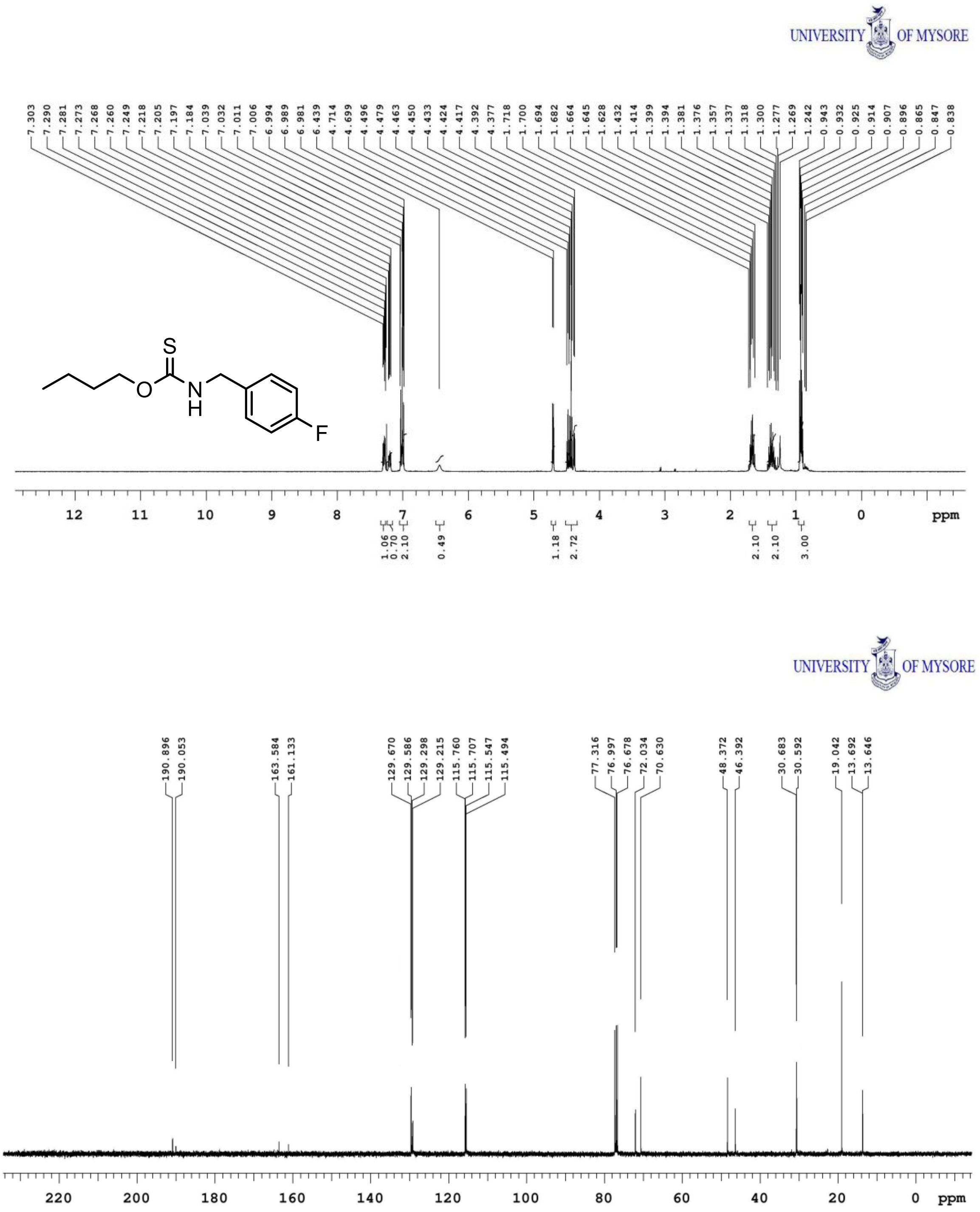

Figure S9: ^{1}H and ^{13}C NMR spectra of **4i**.

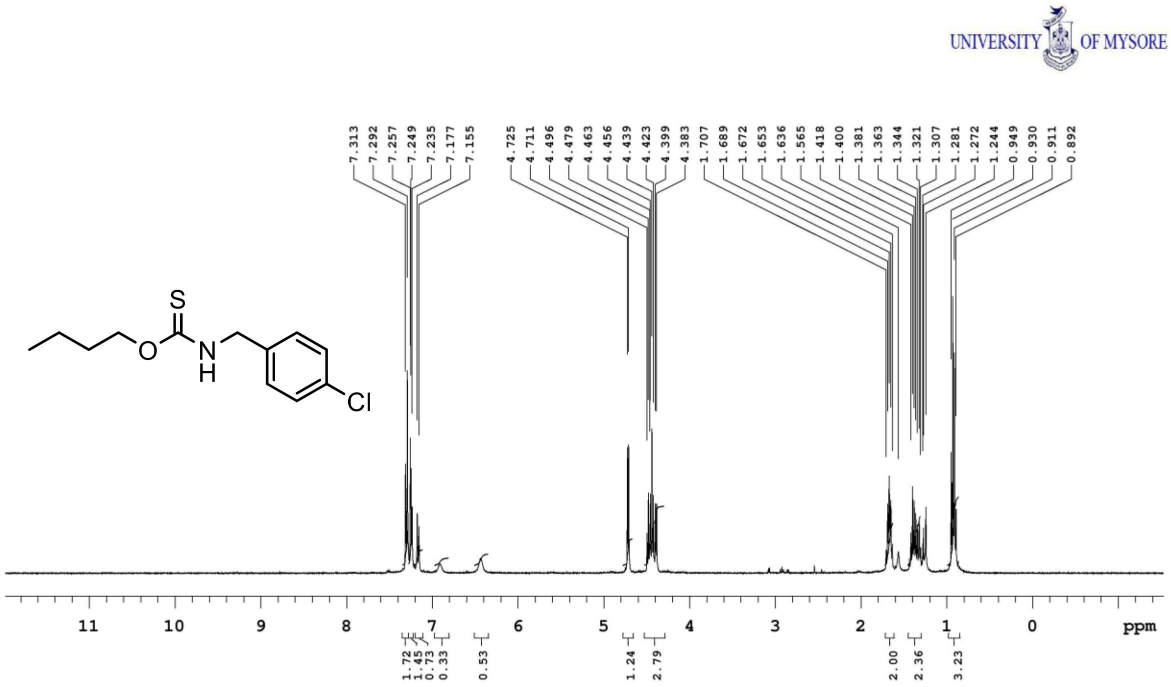

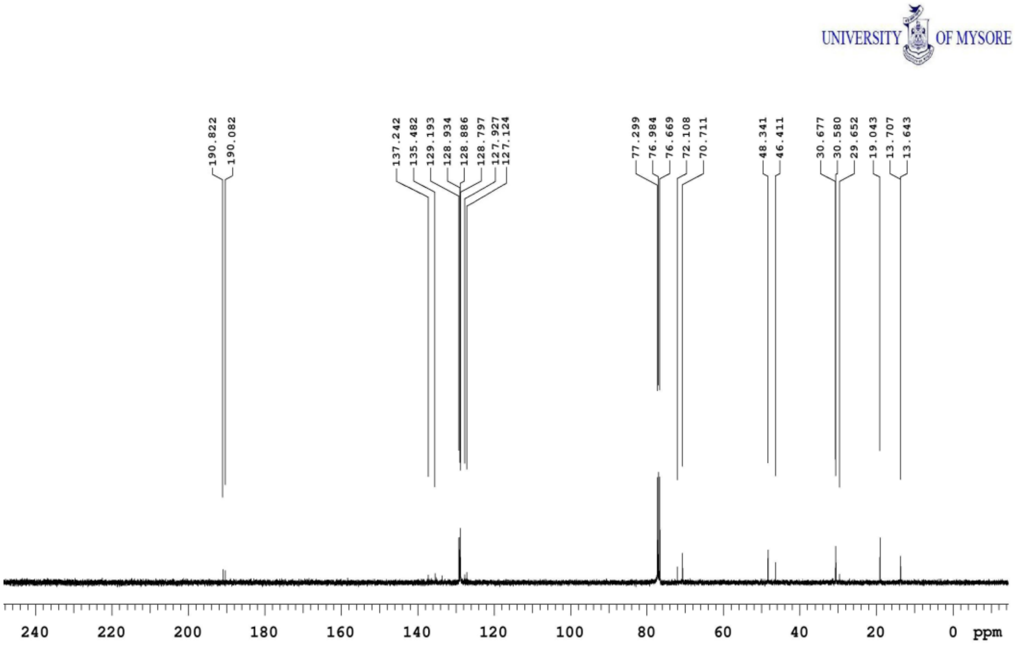

Figure S10: ^{1}H and ^{13}C NMR spectra of **4j**.

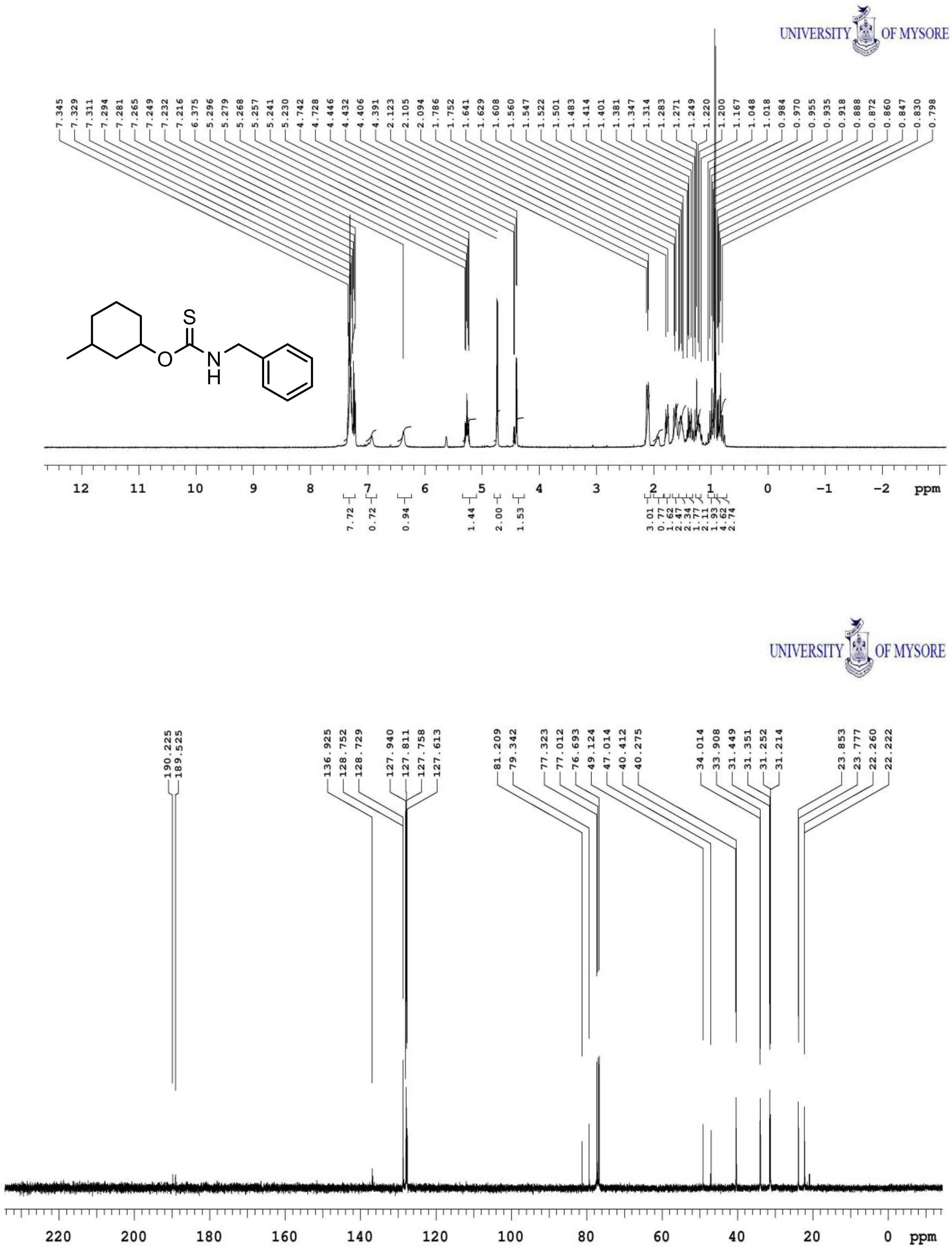

Figure S11: ^{1}H and ^{13}C NMR spectra of **4k**.

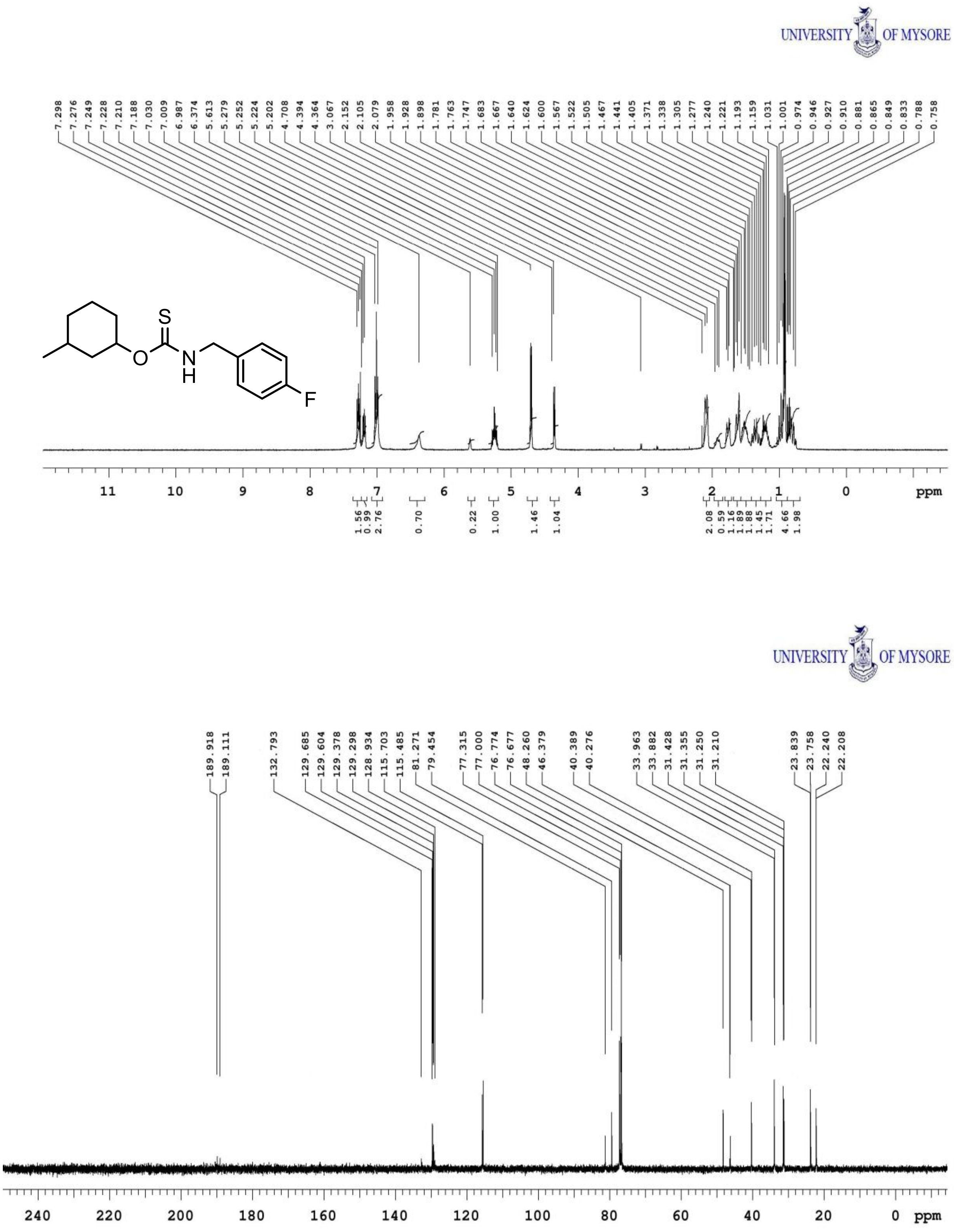

Figure S12: ^1H and ^{13}C NMR spectra of **4l**.

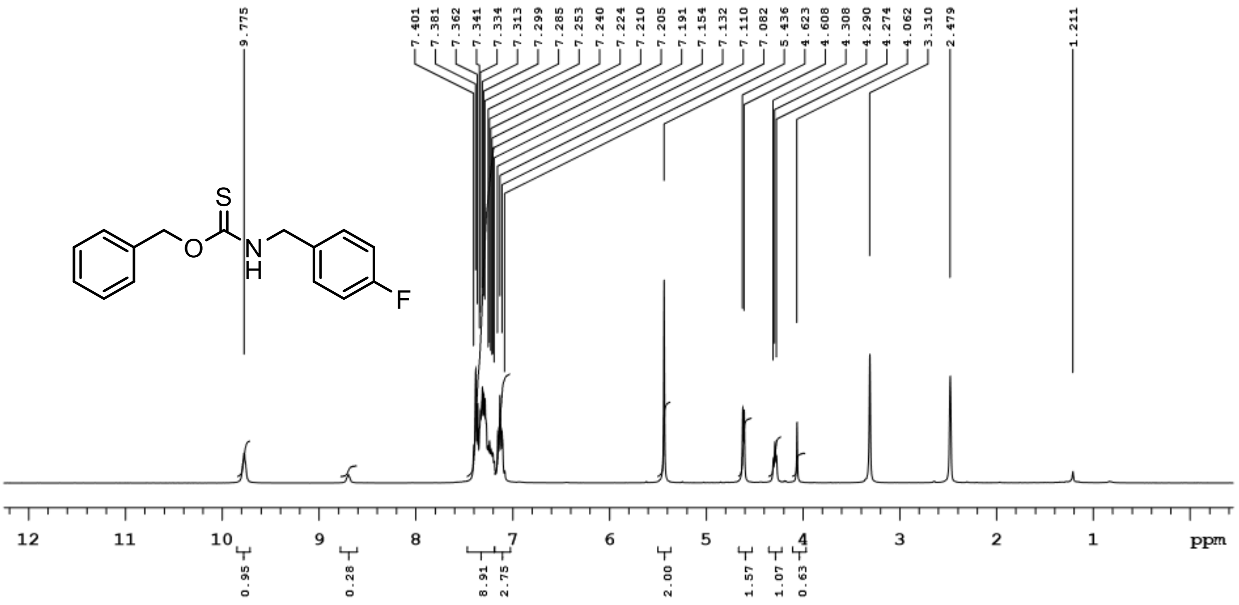

^{1}H NMR spectrum of **4c** in DMSO-d$_6$ under normal conditions.

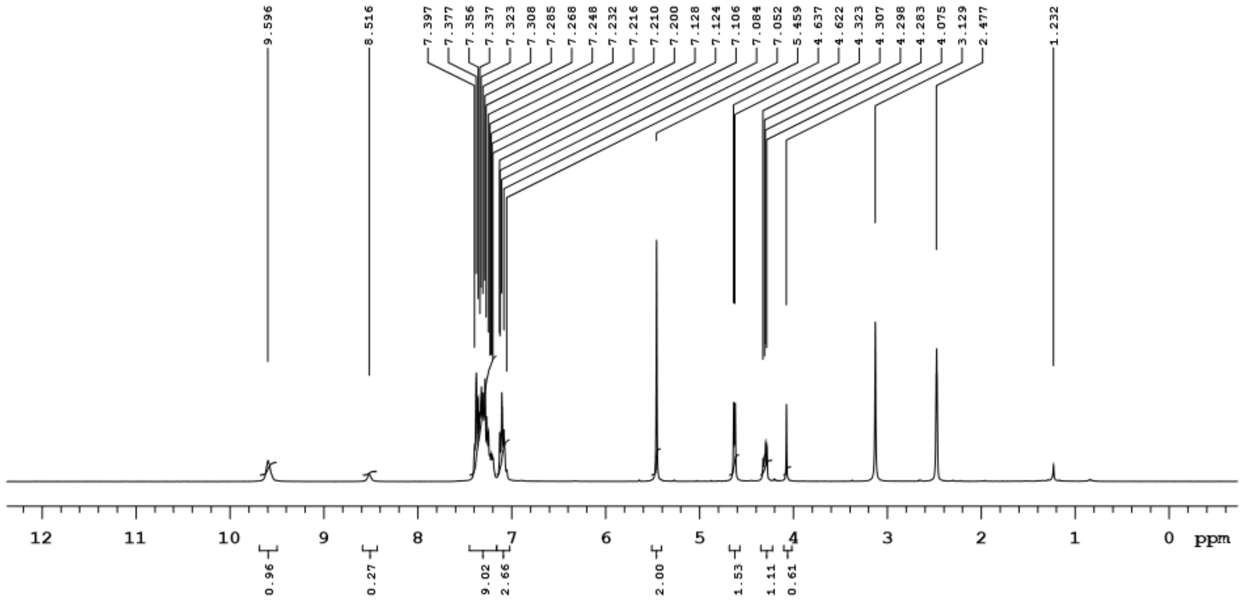

Figure S13: ^{1}H spectrum of **4c** in DMSO-d_6 after heating to 60 °C.

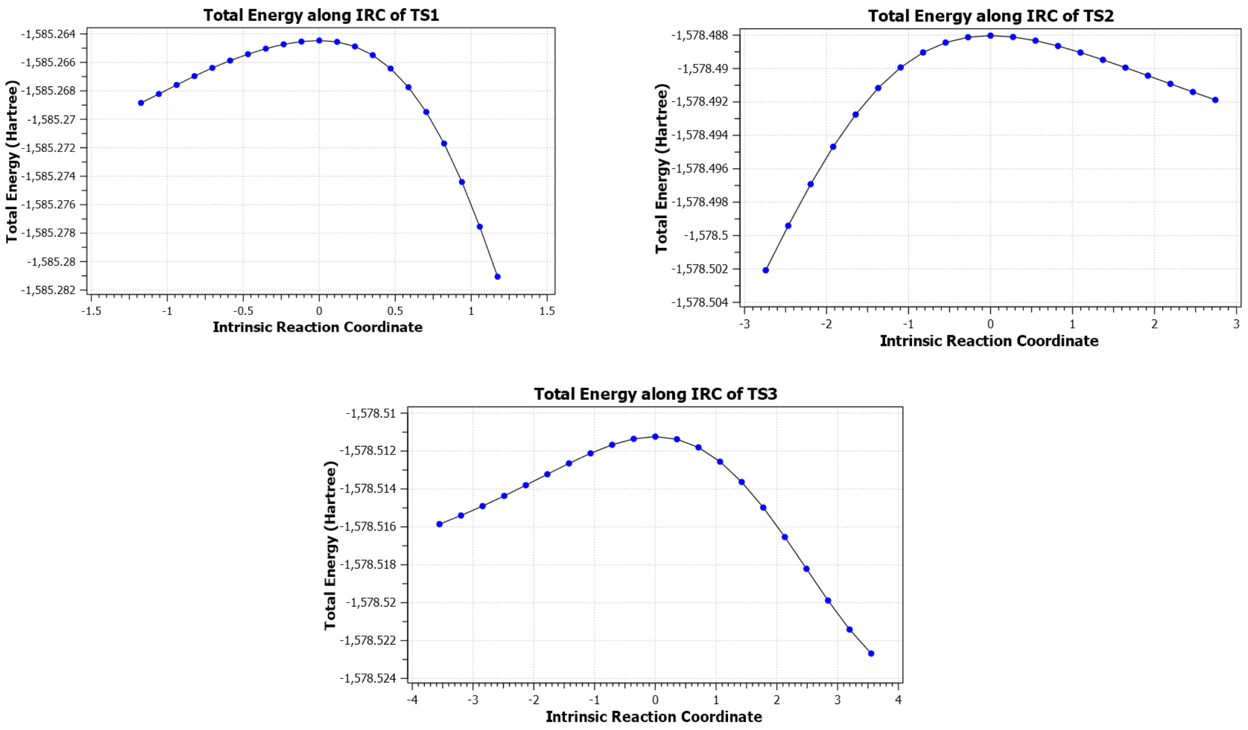

Figure S14: Total energies (Hartrees) along the intrinsic reaction coordinates (IRC) calculated at the HF/6-31G(d) level of theory in the gas phase for the two transition states of the proposed mechanism (see Scheme 2, Figures 4 and 5 in the main text).

Table S3: Relative electronic energies (E_{ele}), activation energies ($E_{thermal}$), enthalpies (H), free energies (G) in kJ mol^{-1} calculated at the B3LYP/ 6-311++G(d,p) level of theory in DMF solvent system for the species involved in the proposed mechanism.

Species	E_{ele}	$E_{thermal}$	H	G
Reactants	0.0	0.0	0.0	0.0
TS1	45.2	42.2	42.2	57.9
Int1	-43.2	-29.8	-29.8	-7.7
TS2	-5.4	5.3	5.3	44.5
Int2	-65.0	-46.1	-46.1	-3.4
TS3	-37.6	-21.4	-21.4	16.7
Int3	-63.1	-44.7	-44.7	-22.0

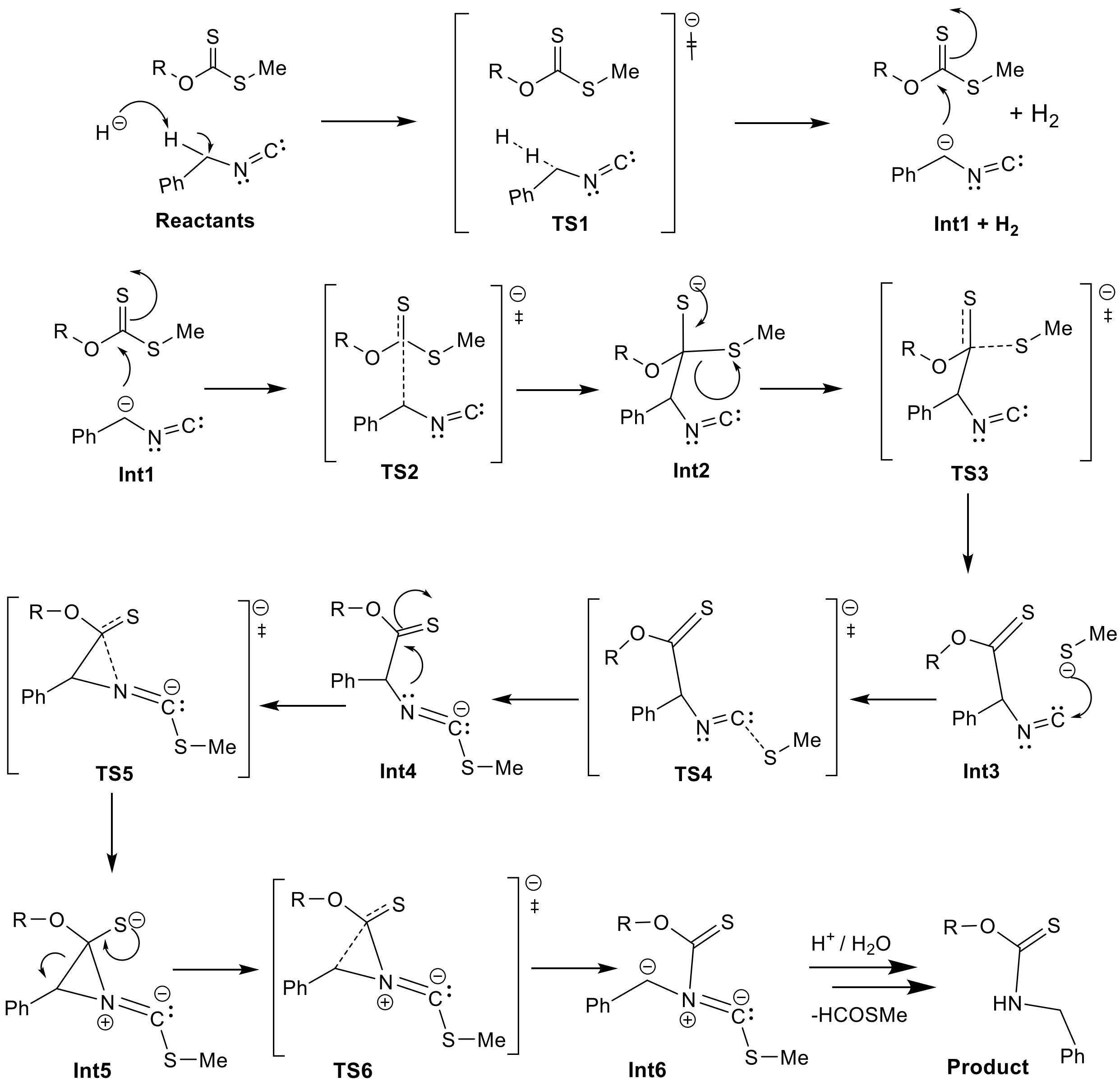

Scheme S1: The alternative mechanism previously considered [Reference SI4], involving the initial nucleophilic addition of a preformed benzylic α-carbanion to the thiocarbonyl group of the xanthate.

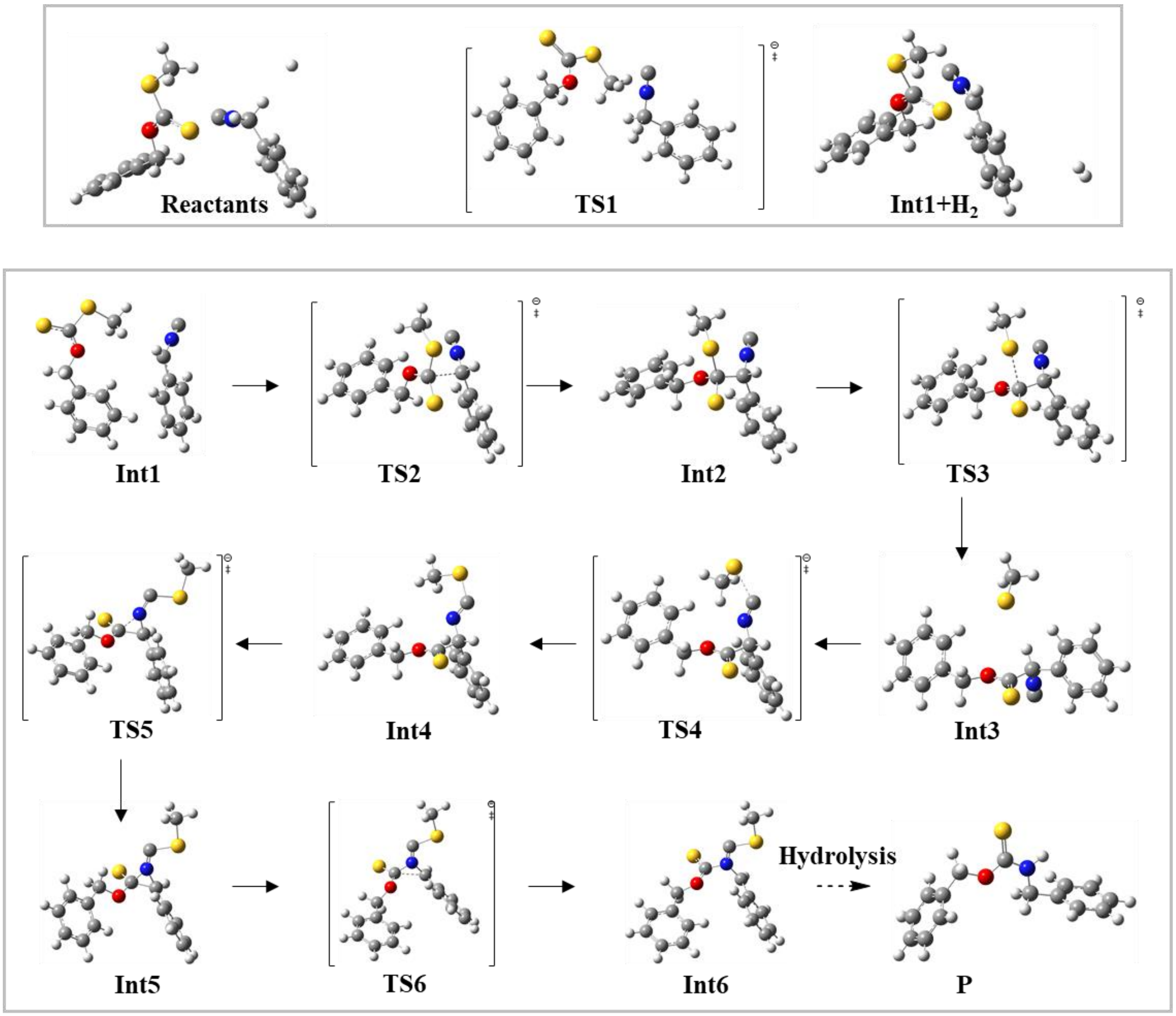

Figure S15: The optimized geometries of reactants, transition states, intermediates, and the product of the proposed reaction mechanism at the HF/6-31G(d) level of theory in gas phase (note: the reaction leading to the proton abstraction from the benzylic CH_2 group to form the Intermediate **Int1** via the transition state **TS1** is shown on the first line. For the subsequent steps, i.e., **Int1**–**Int6**, the geometry optimizations were performed without including the H_2. However, the energy of the H_2 has been added to the energy of each species shown in Figure S16 to correctly compare the relative energies for both proposed mechanisms).

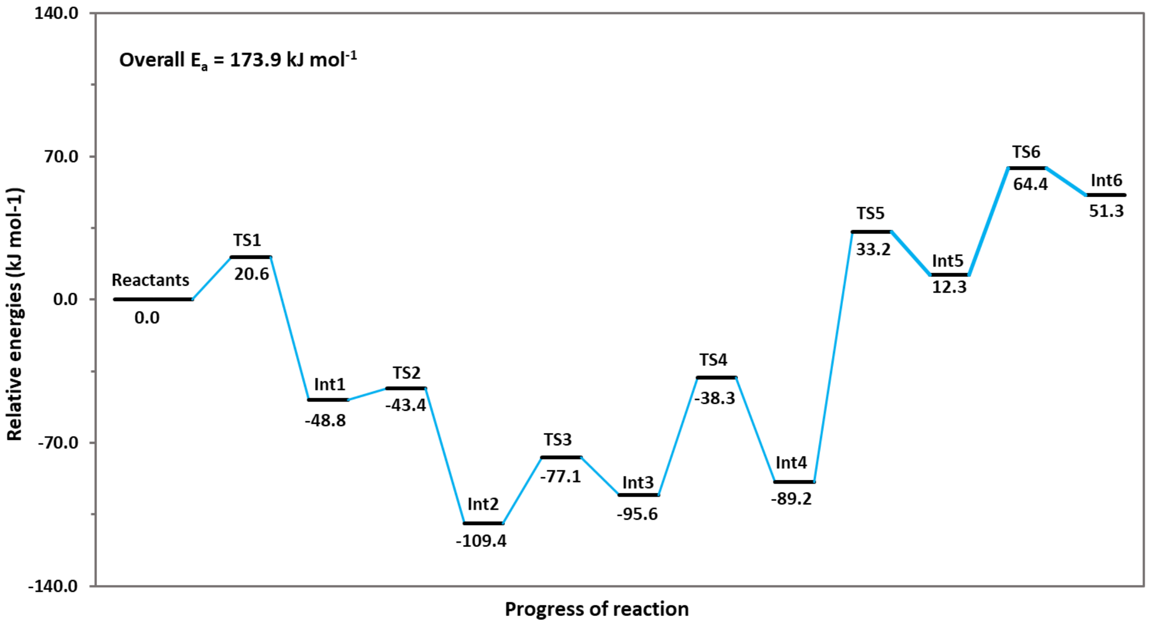

Figure S16: Relative energies of reactants, transition states (**TS1**–**TS6**), and intermediates (**Int1**–**Int5**) of the previously proposed reaction mechanism [Reference SI4] computed (this work) at the B3LYP/6-311++G(d,p) level of theory in a DMF solvent system.

References

[SI1] Bruker, APEX2 and SAINT. Bruker AXS Inc, Madison, Wisconsin, USA 2009.

[SI2] (a) Sheldrick, G. M. Acta. Cryst. **2008**, A64, 112-122. (b) Sheldrick, G. M. ActaCryst. **2015**, C71, 3-8.

[SI3] *Gaussian 09*, Revision C.01, Frisch, M. J.; Trucks, G. W.; Schlegel, H. B.; Scuseria, G. E.; Robb, M. A.; Cheeseman, J. R.; Scalmani, G.; Barone, V.; Mennucci, B.; Petersson, G. A.; Nakatsuji, H.; Caricato, M.; Li, X.; Hratchian, H. P.; Izmaylov, A. F.; Bloino, J.; Zheng, G.; Sonnenberg, J. L.; Hada, M.; Ehara, M.; Toyota, K.; Fukuda, R.; Hasegawa, J.; Ishida, M.; Nakajima, T.; Honda, Y.; Kitao O.; Nakai, H.; Vreven, T.; Montgomery Jr. J. A.; Peralta, J. E.; Ogliaro, F.; Bearpark, M.; Heyd, J. J.; Brothers, E.; Kudin, K. N.; Staroverov, V. N.; Kobayashi, R.; Normand, J.; Raghavachari, K.; Rendell, A.; Burant, J. C.; Iyengar, S. S.; Tomasi, J.; Cossi, M.; Rega, N.; Millam, J. M.; Klene, M.; Knox, J. E.; Cross, J. B.; Bakken, V.; Adamo, C.; Jaramillo, J.; Gomperts, R.; Stratmann, R. E.; Yazyev, O.; Austin, A. J.; Cammi, R.; Pomelli, C.; Ochterski, J. W.; Martin, R. L.; Morokuma, K.; Zakrzewski, V. G.; Voth, G. A.; Salvador, P.; Dannenberg, J. J.; Dapprich, S.; Daniels, A. D.; Farkas, Ö.; Foresman, J. B.; Ortiz, J. V.; Cioslowski, J.; Fox, D. J. Gaussian, Inc., Wallingford CT, 2009.

[SI4] *Beilstein Arch.* **2019**, 201926. doi:10.3762/bxiv.2019.26.v1]
